# Evolution of a Cellular Immune Response in *Drosophila*: A Phenotypic and Genomic Comparative Analysis

**DOI:** 10.1093/gbe/evu012

**Published:** 2014-01-18

**Authors:** Laura Salazar-Jaramillo, Angeliki Paspati, Louis van de Zande, Cornelis Joseph Vermeulen, Tanja Schwander, Bregje Wertheim

**Affiliations:** ^1^Evolutionary Genetics, Centre for Ecological and Evolutionary Studies, Groningen University, The Netherlands; ^2^Department of Ecology and Evolution, Biophore, University of Lausanne, Switzerland

**Keywords:** comparative genomics, host–parasitoid interactions, innate immunity, hemopoiesis

## Abstract

Understanding the genomic basis of evolutionary adaptation requires insight into the molecular basis underlying phenotypic variation. However, even changes in molecular pathways associated with extreme variation, gains and losses of specific phenotypes, remain largely uncharacterized. Here, we investigate the large interspecific differences in the ability to survive infection by parasitoids across 11 *Drosophila* species and identify genomic changes associated with gains and losses of parasitoid resistance. We show that a cellular immune defense, encapsulation, and the production of a specialized blood cell, lamellocytes, are restricted to a sublineage of *Drosophila,* but that encapsulation is absent in one species of this sublineage, *Drosophila sechellia*. Our comparative analyses of hemopoiesis pathway genes and of genes differentially expressed during the encapsulation response revealed that hemopoiesis-associated genes are highly conserved and present in all species independently of their resistance. In contrast, 11 genes that are differentially expressed during the response to parasitoids are novel genes, specific to the *Drosophila* sublineage capable of lamellocyte-mediated encapsulation. These novel genes, which are predominantly expressed in hemocytes, arose via duplications, whereby five of them also showed signatures of positive selection, as expected if they were recruited for new functions. Three of these novel genes further showed large-scale and presumably loss-of-function sequence changes in *D. sechellia*, consistent with the loss of resistance in this species. In combination, these convergent lines of evidence suggest that co-option of duplicated genes in existing pathways and subsequent neofunctionalization are likely to have contributed to the evolution of the lamellocyte-mediated encapsulation in *Drosophila*.

## Introduction

The evolution of immune systems is driven by the large diversity of parasites that organisms are exposed to. The ongoing selection pressure is at the root of the extensive variation underlying many of the genes in the immune defense pathways (Christophides et al. 2002; Nielsen et al. 2005; Sackton et al. 2007; Obbard et al. 2009). However, immune defense pathways also comprise elements that are highly conserved across multicellular organisms, such as Toll receptors that function in innate immunity of both vertebrates and invertebrates (Kimbrell and Bruce 2001). Conservation may be expected for genes involved in multiple processes or genes that occupy key positions in interaction networks, because increased connectivity can generate greater constraints on protein structure (Fraser et al. 2002; but see also Kopp and McIntyre 2010). With the availability of genome sequences of related species and the tools to investigate genome changes, tackling the complexity of the evolutionary history of the immune systems has become possible.

Insects are ideal for studying the evolution of the immune response, because their immune system is relatively simple compared with vertebrates yet potent and multifaceted. Just as all invertebrates, they rely solely on innate immunity (Lemaitre and Hoffman 2007). This innate immunity system consists of two interacting components, a humoral component, involving the release of molecules such as antimicrobial peptides, and a cellular component, involving the differentiation of several specialized cell groups. Both humoral and cellular components are activated after an immune challenge, but the reaction cascades induced by microparasites (e.g., bacteria and fungi) and macroparasites (e.g., parasitic wasps) result in substantially different defensive responses, because micro- and macroparasites differ in size and biochemical composition (important for recognition), and they require different mechanisms to be eliminated or disarmed (Lemaitre and Hoffman 2007).

During the humoral response, surface proteins of pathogens are detected by pattern recognition proteins of the host, which activate two primary signal transduction pathways, the Toll and IMD pathways. A third immunity pathway, RNAi, is directed against viruses. These pathways trigger the transcription and release of antimicrobial peptides and other effector proteins, which directly attack parasites (Lemaitre and Hoffman 2007). Comparative genomic studies in the genus *Drosophila* revealed divergent evolutionary patterns for different groups of humoral immune genes. Most genes in the signal transduction pathways occur as single orthologous copies in each species’ genome and are highly conserved, whereas genes encoding pattern recognition and effector proteins have diversified rapidly across species (Sackton et al. 2007). This diversification has been interpreted as the result of a coevolutionary process with the parasites interacting with the hosts’ immune response (Obbard et al. 2009). Genes encoding recognition proteins diversified mainly by accumulating coding mutations, whereas genes encoding effector proteins diversified primarily through duplication (Sackton et al. 2007; Waterhouse et al. 2007).

The cellular response involves epithelial barriers, as well as specialized blood cells. Different types of blood cells (collectively called hemocytes) mediate defensive processes, whereby the hemocytes can change in morphology and abundance after infection (Gillespie and Kanost 1997; Krzemien et al. 2010). In *Drosophila melanogaster*, the three most common blood cell types are plasmatocytes, lamellocytes, and crystal cells. Plasmatocytes perform phagocytosis of bacteria and other small pathogens; lamellocytes form a layer around large foreign bodies; and crystal cells store the precursors of the melanin that is deposited on invading pathogens (Fauverque and Williams 2011). In unchallenged *Drosophila* larvae, lamellocytes are typically absent or detectable only in very low densities among the circulating hemocytes, whereas parasitization by macroparasites can (strongly) induce the proliferation and differentiation of lamellocytes from both the lymph glands (the hematopoietic organ in *Drosophila*) and from circulating undifferentiated hemocytes (for simplicity, we may refer to this induced proliferation and differentiation as the “production” of lamellocytes). The main cellular immunity pathways are the Toll, JAK/STAT, and JNK pathways (Meister 2004), but it is not clear whether selection pressures imposed by parasites may have driven diversification patterns in these pathways similar to those found in the humoral pathways.

There are at least two reasons why the evolutionary patterns found for the humoral response may not be representative of the cellular response. First, the process of producing and releasing (humoral) molecules is fundamentally different from the process of differentiating and proliferating specialized cells. Second, expression experiments indicated that the genes differentially expressed after microbial infection differ considerably from those differentially expressed under parasitoid attack, and the humoral pathways RNAi and IMD do not show up-regulation under wasp attack (Wertheim et al. 2005; Schlenke et al. 2007). These substantial differences may be the consequence of different evolutionary dynamics for the humoral and cellular innate immune responses.

**Fig. 1.—:**
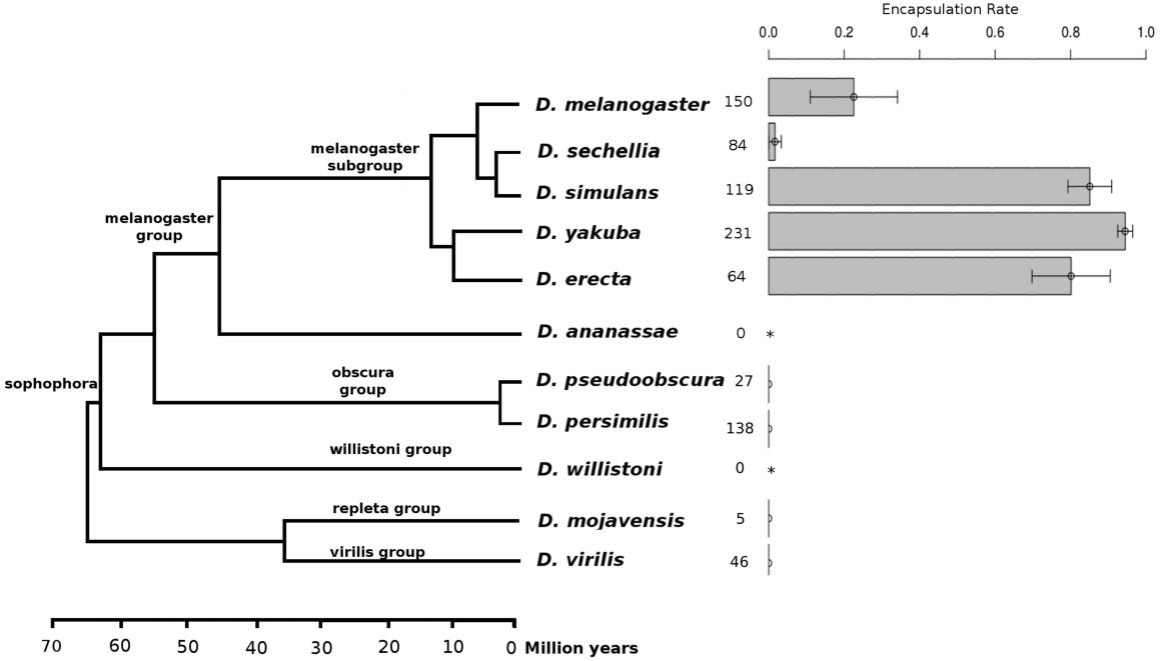
Encapsulation rate of *Drosophila* species against *Asobara tabida.* Mean and standard error of the encapsulation rate (ER), defined as ER = *c*/(*c* + *w*), where *c* is the number of adult flies carrying a capsule and *w* the number of emerged wasps. For each species, we also provide the numbers of parasitized larvae (*c* + *w*). In some species, *A. tabida* did not develop (asterisks), and other species showed very high mortality rates after parasitization (see supplementary fig. S7, Supplementary Material online, for more detailed estimations of rates of parasitism, mortality, and resistance). The phylogeny is adapted from Singh et al. (2009).

In this study, we investigate the genomic changes associated with the evolution of cellular immunity in the *Drosophila* genus, specifically the encapsulation response against parasitoids. Parasitoids are insects that lay eggs in or on other insects, and kill their host during development (Godfray 1994). To neutralize a parasitoid egg by encapsulation, the host has to detect the egg, surround it with multiple layers of hemocytes, and fully melanize it (from hereon this process is referred to as “encapsulation ability”). When the melanotic encapsulation response is not fast or strong enough, the developing wasp kills the host (Strand and Pech 1995). Within the *Drosophila* genus, there is large variation in encapsulation ability, from completely absent in some species to high in others. The hemocyte load of the host (constitutive or induced) was shown to correlate with encapsulation success rates in species of the *melanogaster* subgroup (Eslin and Prevost 1998). The ability to encapsulate does not (only) depend on the natural exposure to parasitoids, because some species in the *obscura* group are natural hosts of parasitoid wasps but completely deficient for encapsulation ability (Eslin and Doury 2006; Havard et al. 2009). To investigate the genomic basis of the ability to encapsulate, we conducted parasitization experiments and a genomic characterization across a broad taxonomic range of 11 sequenced *Drosophila* species (fig. 1) (Drosophila 12 Genomes Consortium 2007). Focusing on genes that have been shown to be involved in hemopoiesis (Zettervall et al. 2004; Williams 2007; Stofanko et al. 2010; Avet-Rochex et al. 2010) and on genes differentially expressed after parasitoid attack in *D. melanogaster* (Wertheim et al. 2005; Schlenke et al. 2007), we identified orthologs in all 11 species and studied the divergence in terms of both 1) presence–absence and 2) sequence variation of protein coding genes.

## Materials and Methods

### Species Strains

The 11 *Drosophila* strains used in this study were all genome project strains from the *Drosophila* Stock Center (San Diego University) (Drosophila 12 Genomes Consortium 2007) (supplementary table S1, Supplementary Material online). Flies were reared at 20 °C under a dark:light regime of 12:12 and 50% relative humidity in quarter-pint bottles containing 30 ml standard medium (26 g dried yeast, 54 g sugar, 17 g agar, and 13 ml nipagine solution per liter), supplemented with a small piece of banana. The parasitoid strain of *Asobara tabida* was originally collected in Sospel, France, and has been maintained on *D. subobscura* at 20 °C under a dark:light regime of 12:12. It has a moderately to high virulence and produces so-called “sticky eggs” that can adhere to host tissue to evade full encapsulation. The parasitoid strain of *A. citri* was collected in Ivory Coast and has been maintained on *D. melanogaster* at 25 °C under a dark:light regime of 12:12.

### Encapsulation Assay

We tested the encapsulation ability of the 11 *Drosophila* species (Drosophila 12 Genomes Consortium 2007) against two different parasitoid wasp species from the *Asobara* genus, *A. tabida* and *A. citri*. Fifty second-instar larvae (∼48 h after egg laying at 25 °C) were exposed to either two wasp females of *A. tabida* or one female of *A. citri*. We used two wasps for *A. tabida* to increase parasitization rates, whereas for *A. citri*, single females achieved high parasitization rates. All infections were carried out at 20 °C on a Petri dish of 70-mm diameter filled with standard medium. Typically, eight Petri dishes with 50 larvae were examined, whereas for some species, only four (due to culturing difficulties). Wasps were removed 3 days later, and five larvae per Petri dish were dissected to confirm parasitization by the wasp (except for the *D. mojavensis*, for which dissections were not carried out because the amount of eggs laid and larvae developed was too small). We recorded superparasitism in our dissection assays, which was occasionally found but did not differ substantially among host species nor affected the results qualitatively (data not shown). The rest of the larvae were allowed to complete development, and the number of emerging flies with capsule and wasps was recorded for each Petri dish. Capsules in adult flies were recorded by squashing the adult between two glass slides under a stereo microscope. Each Petri dish was considered an independent replicate. We used a Generalized Linear Model (glm) implemented in R 2.15.1 (R Development Core Team 2008) to analyze the number of wasps and flies with capsule (ratio) that emerged (binding the variables in a matrix) and considering fly species (FlySp) as explanatory variable. We used Binomial error distribution (logit-link function) and a quasibinomial distribution to correct for overdispersion.
(1)
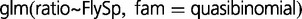



To test the contribution of the explanatory variable to the model, we used an analysis of deviance for generalized model fit using *F*-tests.

### Lamellocyte Identification

To assess lamellocyte production, we exposed 50 second instar larvae to *A. tabida* and observed the oviposition behavior of the wasps. We collected only larvae for which parasitization was recorded (the wasp spent at least 10 s ovipositing). We also collected larvae that were not exposed to wasps as control. At 96 h after parasitization, that is, when the larvae were in the third instar stage, we pricked five larvae with a fine needle and collected their pooled hemolymph. We diluted 1 µl of the pooled hemolymph into 7 µl of Ringer’s solution (13.6 g KCl, 2.7 g NaCl, 0.33 g CaCl_2_, and 1.21 g tris solution per liter) to fill a hemocytometer slide Neubauer Improved (0.1 mm depth). We repeated this at least five times per species. We observed the samples at 40× objective magnification under a phase-contrast microscope. Lamellocytes can be recognized by their flat shape compared with other blood cells (supplementary fig. S1, Supplementary Material online). Pictures were made with a Moticam 2000 (2M pixel) camera.

### Melanization

To test the ability to melanize after injury, five second instar larvae were pricked with a fine needle and scored for the presence of a black spot after 2 and 4 h.

### Candidate Genes

The set of candidate genes we analyzed was composed of 144 protein-coding genes, 35 with a GeneOntology annotation of “hemocyte differentiation,” “hemocyte proliferation,” or “regulation of hemocyte differentiation” in Flybase (version FB2012 04) (McQuilton et al. 2012) and 109 protein-coding-genes based on the studies by Wertheim et al. (2005) and Schlenke et al. (2007), and compiled in Kraaijeveld and Wertheim (2009). Both studies are genome-wide expression data from microarray experiments of *Drosophila* larvae parasitized by wasps: the first study by *A. tabida* and the second by *Leptopilina heterotoma* and *L. boulardi*.

### Orthologous Groups and Homology Categories

Orthologs to the *D. melanogaster* candidate genes in the remaining 10 *Drosophila* species were found using OrthoMCL (Li et al. 2003). This algorithms uses Blast similarity score to find best reciprocal hits between complete genomes (we used the default cut-off value, i.e., 10^−^^5^). Proteins were subsequently clustered into within-species best reciprocal hits (inparalogs) and between-species best reciprocal hits (outparalogs). Outparalogs are those proteins that share orthologs inside and across species and represent ancient duplicates (predating speciation). The distinction between in- and outparalogs allows the differentiation of recent from ancient paralogs. We used these clusters of orthologs to detect the pattern of gene presence–absence. We used three general homology classes: “single copy ortholog” (SCO) for genes that have exactly one copy in each species, “paralog” (PAR) for genes with multiple orthologs in more than two species, and “lineage restricted” (LR) for those genes present in a (monophyletic) subset of the lineage. Recent paralogs were included in the lineage-restricted class, because they constitute lineage-specific expansions. The clusters of orthologous groups were aligned using ClustalW 2.0.10 (Larkin et al. 2007). Functional domains were visualized in Pfam, a database of protein families (Punta et al. 2012).

### Phylogenetic Analysis

For the recent duplications (inparalogs), we analyzed the protein tree to distinguish the new copy from the old. We used ModelGenerator (Keane et al. 2006) to choose the best substitution model for each particular cluster. Then we reconstructed the phylogeny with PhyML v3.0 (Guindon et al. 2010) and calculated the bootstrap values of each branch 100 times. Phylogenetic trees were made with PHYLIP (Felsenstein 2005) and drawn with FigTree (http://tree.bio.ed.ac.uk/software/figtree/, last accessed January 30, 2014).

### Immune Classification

The 35 genes annotated in Flybase with a function in hemocyte differentiation and proliferation were classified as “hemopoiesis.” For the 109 genes from the genome-wide expression study, we followed the immunological categories in (Sackton et al. 2007; Waterhouse et al. 2007): recognition, signaling, and effector. We included an extra functional category, namely serine proteases, which is analogous to the “modulator category” in (Waterhouse et al. 2007). “Recognition” refers to putative pattern-recognition receptors and proteins involved in binding; “signaling proteins” are those that have been characterized in immune signal transduction pathways, namely Toll, Jak/Stat, IMD, and JNK; and “effectors” are antimicrobial peptides, phenoloxidases, and intermediates in the melanin production.

### Positive Selection

We used PAML 4.4 (Yang 2007), a package of programs for analyzing sequences using maximum likelihood. This program is based on the phylogenetic comparison of synonymous (d*S*) and nonsynonymous (d*N*) substitution rates, expressed in the ratio: ω = d*N*/d*S*. We applied a maximum likelihood test in two sets of models that allow ω to vary per position: one nearly neutral (M1a) model, where ω is between (0,1) and against a model of positive selection, where ω is between (0,2). Models M7 and M8 use the same concept but for a continuous beta distribution. We calculated two times the difference in likelihoods between the corresponding nested models (i.e., M1 vs. M2, and M7 vs. M8), obtained the *P* value from a χ^2^ distribution with two degrees of freedom (Yang 2007), and corrected for multiple testing using false discovery rate (FDR) implementing a bootstrap method. This analysis was applied only to branches that had one orthologous gene copy in each species. For instance, in the big family of Tep, we applied the test independently to *TepI*, *TepII*, *TepIII*, and so on, rather than to the whole orthologous group. Orthologous groups with multiple copies in one species (i.e., paralogs) were left out of the analysis.

### RT-qPCR

To compare gene expression between parasitized and control larvae of *D. melanogaster*, *D. simulans,* and *D. sechellia*, reverse transcription quantitative PCR (RT-qPCR) was performed on second instar larvae parasitized by *A. tabida* (we used the inbred “TMS” line derived from the *A. tabida* strain collected in Sospel) and nonparasitized control larvae (collected in parallel). For each biological replicate, total RNA was extracted from pools of five larvae that were collected at 5 h and 50 h after parasitoid attack. These time points were chosen based on the expression profiles of our three target genes (see later) in an earlier microarray experiment (Wertheim et al. 2005). RNA was extracted and purified using a combination of Trizol (Invitrogen, Carlsbad, CA) and RNeasy (Qiagen, Hilden Germany), according to the manufacturer’s instructions. Tissue homogenization and cell lysis were performed using a pestle in 1 ml Trizol, and RNA purification on the RNeasy columns included genomic DNA digestion with DNAseI (Qiagen, Hilden, Germany). cDNA was synthesized from 10 µl RNA, using RevertAid RT (Fermentas). Primers were designed on exon–exon boundaries whenever possible, using the Perlprimer software (Marshall 2004). A common primer set for all species could be designed for the two endogenous reference genes (*Act 5C* and *fd68A*) and two target genes (*IM1* and *PPO3*). The high divergence of *TepI* necessitated a specific primer set for each species (See supplementary table S5, Supplementary Material online, for the primers). Primers were checked for linear amplification efficiencies and optimized. The cDNA template for one of the reference genes (*Act 5C*) had to be diluted (5 h: 50×; 50 h: 100×) to avoid formation of secondary structures. The qPCRs were performed in total volumes of 25 µl per reaction in an Applied Biosystems 7300 Real Time PCR System, using Absolute QPCR SYBR Green ROX mix (Abgene, Hamburg, Germany). Data were analyzed, using the algorithm implemented in the statistical package qpcR (version 1.3-6) (Ritz and Spiess 2008). The median of three technical replicates was obtained for each of five biological samples. Quantification was based on the window-of-linearity method that incorporates individual PCR efficiencies for each sample. The expression of the target genes per biological replicate was standardized to the geometric mean of the two reference genes (Vandesompele et al. 2002). Statistical differences were estimated for the fold-changes between parasitized and control larvae using the permutation method for error estimation. All scripts were run using Python 2.7.3 and R 2.15.1, and are available upon request.

## Results

### Phenotypic Characterization: Only Species of the *melanogaster* Subgroup Show Encapsulation Ability and Produce Lamellocytes

For the phenotypic characterization, we used 11 *Drosophila* species, of which the genomes are publicly available. These species come from different geographical ranges, some being cosmopolitan such as *D. melanogaster* and *D. simulans*, some with large geographical ranges such as *D. ananassae* (Asia and Pacific), *D. yakuba* (Africa), *D. virilis* (Holartic), *D. pseudoobscura*, *D. persimilis,* and *D. willistoni* (America), and some species with (very) limited distributions such as *D. erecta* (west Africa), *D. mojavensis* (Mojave desert), and *D. sechellia* (Seychelles Islands) (Powell 1997; Singh et al. 2009). Species of *Drosophila* are known to act as host for a variety of larval and pupal parasitoids, with members of the genera *Asobara* (Hymenoptera: Braconidae) and *Leptopilina* (Hymenoptera: Figitidae) being the most common threat across the world (Carton et al. 1986; Fleury et al. 2009). We used *A. tabida* to test the encapsulation ability of the *Drosophila* species, as this species has an evasive virulence mechanism (some strains, including ours, produce “sticky eggs”) that does not require specificity in the host defenses (Eslin and Prevost 2000). The *A. tabida* distribution is holartic, and it has been found as natural parasitoid of some species of the *melanogaster* and *obscura* groups in Europe and America (Eslin and Prevost 2000; Kraaijeveld and van Alphen 1993).

The proportion of larvae that successfully encapsulated eggs of the parasitoid wasp *A. tabida* varied significantly among *Drosophila* species (glm, *F* = 53.37, DF1 = 8, DF2 = 51, *P* < 2.2e − 16) (fig. 1). To ensure that the lack of resistance in some *Drosophila* species was not due to a lack of coevolutionary history with the holartic *A. tabida* (e.g., a complete lack of species interaction could result in failure to recognize or respond to the immune challenge), we also tested the encapsulation ability against an African *Asobara* species, *A. citri*, and screened the literature for additional information. *Drosophila* species unable to encapsulate *A. tabida* were also unable to encapsulate *A. citri* or two parasitoid species from the genus *Leptopilina* (table 1). Of all *Drosophila* species tested, only species of the *melanogaster* subgroup, except *D. sechellia* inside this group, showed any encapsulation ability against *A. tabida* (fig. 1).

**Table 1:** Phenotypic Characterization of Cellular Immune Response

*Drosophila* Species	Melanotic Encapsulation against		Lamellocytes	Crystal Cells^a^	Melanization^a^
	*Asobara*^a^	*Leptopilina*^b^			
*melanogaster*	*tabida*	*boulardi*	Yes^a^^,^^c^	Yes	Yes
*simulans*	*tabida*	*boulardi*	Yes^a^^,^^c^	Yes	Yes
*sechellia*	None	None	Yes^a^^,^^c^	Yes	Yes
*yakuba*	*tabida*	*boulardi*	Yes^a^^,^^c^	Yes	Yes
	*citri*	*heterotoma*			
*erecta*	*tabida*	*boulardi*	Yes^a^^,^^c^	Yes	Yes
	*citri*	*heterotoma*			
*ananassae*	None	None	No^a^	Yes	Yes
*persimilis*	None	None	No^d^	Yes	Yes
*pseudoobscura*	None	None	No^d^	Yes	Yes
*willistoni*	None	None	No^a^^,^^e^	Yes	Yes
*mojavensis*	None	None	No^a^	Yes	Yes
*virilis*	None	None	No^a^	Yes	Yes

Note.—The cellular immune response of 11 *Drosophila* species against parasitoids, based on experimental assays and published literature. The first two columns list the encapsulation ability against various tested parasitoids species of two distant genera, *Asobara* and *Leptopilina*. The third and four columns refer to evidence of lamellocyte and crystal cells production, respectively. The last column refers to the ability to initiate a melanization response after injury.

^a^This study.

^b^Schlenke et al. (2007).

^c^Eslin and Prevost (1998).

^d^Havard et al. (2009).

^e^Unusual hemocytes were observed (see supplementary fig. S2, Supplementary Material online).

To further characterize the differences in encapsulation of parasitoids, we investigated two traits that are important for the encapsulation process, the melanization ability and production of lamellocytes. During the dissections of a subset of larvae for each species, we noticed that species unable to encapsulate did not show any signs of melanization around the parasitoid eggs. We verified that all species were able to melanize, independent of the encapsulation process, by pricking the larvae with a fine needle (table 1, supplementary fig. S1, Supplementary Material online). All species did melanize the site of injury, which indicates that the lack of resistance in some species was not due to a general lack of melanization ability.

We confirmed the ability to produce lamellocytes in *D. sechellia*, *D. melanogaster*, *D. simulans*, *D. yakuba,* and *D. erecta* from the *melanogaster* subgroup, and tested *D. annanassae*, *D. willistoni*, *D. mojavensis,* and *D. virilis* outside this group. For the *obscura* group, we relied on the detailed characterization in Havard et al. (2012). Lamellocytes were produced only by species in the *melanogaster* subgroup, whereas species outside this group do either not differentiate lamellocytes at all or only a large type of hemocytes with an unusual morphology, that is, not as flat or big as lamellocytes (see supplementary fig. S2, Supplementary Material online, for details). Lamellocyte production in *Drosophila* therefore appears to be necessary but not sufficient for encapsulation ability, as evidenced by the lack of encapsulation in *D. sechellia*, which produced lamellocytes.

Two additional species (*D. eugracilis* and *D. suzukii*) in the *melanogaster* group and outside the *melanogaster* subgroup have been reported to encapsulate wasp eggs and produce lamellocytes (Schlenke et al. 2007; Kacsoh and Schlenke 2012). More distantly related *Drosophila* species have also been reported to encapsulate parasitoid eggs, yet by means of another type of hemocytes (“pseudopodocytes” in the *obscura* group) (Havard et al. 2012) or without specifying the involved hemocyte types (Streams 1968). Two *Drosophila* species in our assay (*D. willistoni* and *D. ananassae*, fig. 1) seemed to resist parasitoid development through other mechanisms than encapsulation. We confirmed through dissections that both species were parasitized by *A. tabida* but no *A. tabida* eggs developed in this species, whereas *A. citri* could develop but never induced melanotic capsules. This suggests either incompatibility of these two species with *A. tabida* or they evolved a different defense mechanism against (some) parasitoids. The combined information across all studied *Drosophila* species indicates that the ability to defend against parasitoids has been gained and lost repeatedly in the *Drosophila* phylogeny, possibly by means of gaining and losing different immunity components, including different types of hemocytes. Because of the uncertainty in the homology of the encapsulation mechanism for more distant species, we focus on the mechanism found in *D. melanogaster* and close relatives. Our current knowledge indicates that a sublineage inside the *melanogaster* group shows: 1) encapsulation of several parasitoid species mediated by the differentiation of lamellocytes and 2) loss of resistance in *D. sechellia*.

### Comparative Genomics

To associate the striking dichotomy that we found across the 11 *Drosophila* species in both lamellocyte differentiation and encapsulation ability, with changes and variation in their genomes, we applied comparative genomic approaches on a list of “candidate genes.” We explored the genomic variation of genes in hemopoiesis pathways on 35 protein coding genes with GeneOntology annotation of “hemocyte differentiation,” “hemocyte proliferation,” or “regulation of hemocyte differentiation” in Flybase (version FB201204) (Zettervall et al. 2004; Williams 2007; Fauverque and Williams 2011; McQuilton et al. 2012) or identified as inducers of lamellocyte differentiation through lineage tracing studies (Avet-Rochex et al. 2010; Stofanko et al. 2010). Because the genetic mechanisms that induce and regulate the proliferation and differentiation of hemocytes upon parasitization have not been fully elucidated, we also analyzed 109 genes that were previously found to be differentially expressed after parasitoid attack in *D. melanogaster* (Wertheim et al. 2005; Schlenke et al. 2007; Kraaijeveld and Wertheim 2009). Lamellocyte differentiation is strongly induced by parasitoid attack, and therefore, the genome-wide transcriptional response after parasitoid attack can help to identify genes involved in this process. Genes were classified in five immunological categories, partially following (Sackton et al. 2007; Waterhouse et al. 2007; Kraaijeveld and Wertheim 2009): 1) “hemopoiesis,” containing the 35 genes annotated in Flybase with a function in hemocyte differentiation, regulation of differentiation and proliferation; 2) “recognition,” containing putative pattern recognition receptors; 3) “signaling,” containing genes characterized in immune signal transduction pathways (Toll, Jak/Stat, IMD, and JNK); 4) “effectors,” coding for antimicrobial peptides, phenoloxidases and mediators in the melanin production; and 5) “proteases,” containing serine-type endopeptidases with mostly unknown immune function but sometimes referred to as modulators. The full list of analyzed genes and their classification is included in supplementary table S3, Supplementary Material online. A subset of 71 of the total 144 genes have also been reported as part of the humoral response against microparasites or of a more general stress response and were analyzed in a previous comparative genomics study in the same *Drosophila* species (Sackton et al. 2007). The 71 overlapping genes comprise most genes of the hemopoiesis class (24 out of 35) and the recognition class (12 out of 15), and all the 17 genes in the signaling category. In the protease class, only one gene overlapped (out of 45), and in the effector class 17 (out of 32) overlapped. This partial overlap signifies both a shared actuation and regulatory control of humoral and cellular immune responses against macro- and microparasites, as well as substantial differences downstream in the reaction cascades.

### Orthologs

Of the 144 candidate genes, 96 genes fell into the SCO category, which is representative for the proportion of SCO in the *D. melanogaster* genome (≈50%) (Drosophila 12 Genomes Consortium 2007). Paralogs (PAR) and LR genes were found for 22 and 26 proteins, respectively (supplementary table S3, Supplementary Material online).

**Fig. 2.—:**
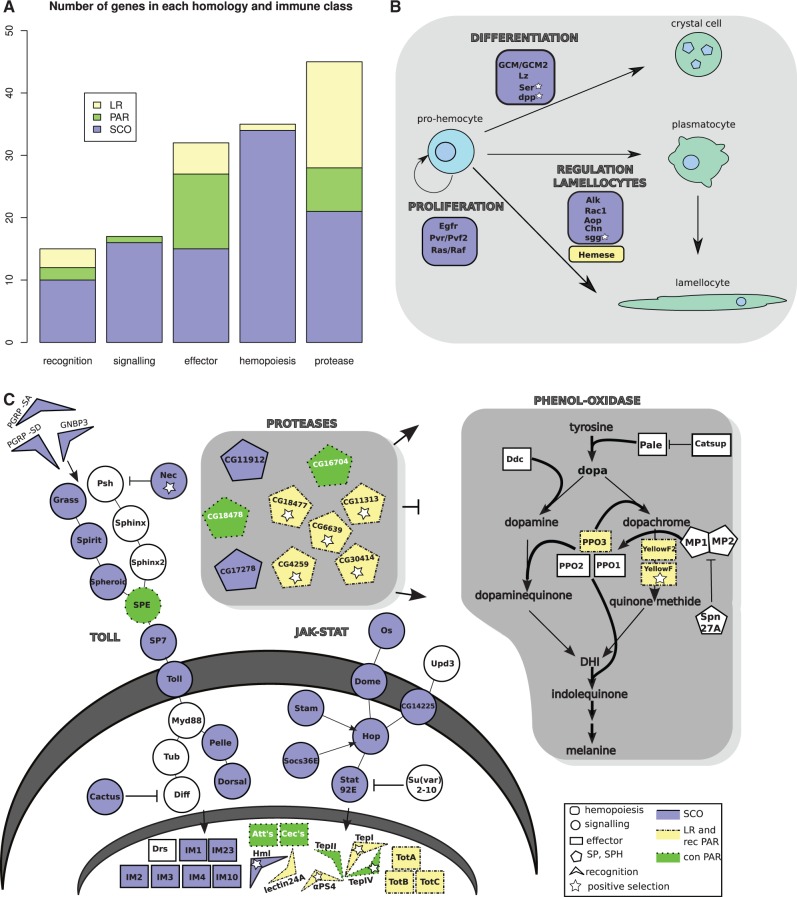
Distribution of proteins in homology and immune categories. (*A*) LR, lineage restricted; PAR, paralogs; SCO, single-copy orthologs. (*B*) Schematic representation of pathways controlling hemopoiesis. Adapted from Meister (2004), Zettervall et al. (2004), and Williams (2007). (*C*) Schematic representation of immune pathways expressed under parasitoid attack. Nonfilled shapes correspond to proteins known to be in the pathway but that were not found to be differentially expressed after parasitoid attack in microarray studies. These genes can still be involved in the encapsulation response. Chemical compounds are shown in plain text. Adapted from Schlenke et al. (2007) and Tang (2009).

The candidate genes in the five immunity categories (recognition, signaling, effectors, proteases, and hemopoiesis) were not uniformly distributed over the three homology classes, SCO, PAR, and LR (χ^2 ^= 45.5, DF = 8, *P* = 3.517e − 06) (fig. 2*A*). A schematic view of the position of the genes in the hemopoiesis and immune pathways is presented in figure 2*B* and *C*. All genes but one in the hemopoiesis class (*Hemese*, a cellular receptor), and most genes in the signaling class (16 out of 17) were SCO. Effector proteins had the largest proportion of PAR (14 out of 32) and proteases, the largest proportion of LR (17 out of 45). The previous comparative study by Sackton et al. (2007) already showed that several of these genes are highly conserved. This is likely caused by strong constraints acting on developmental pathways in general (Artieri et al. 2009; Rebeiz et al. 2011), where changes in gene regulation suffice to create interspecies variation. Our data are consistent with the hypothesis that signaling genes are highly conserved in long-term evolutionary scales, as these genes most likely evolve under strong constraints (Sackton et al. 2007; Waterhouse et al. 2007), and effector genes and proteases diversify mainly through gene duplication (Drosophila 12 Genomes Consortium 2007; Sackton et al. 2007; Waterhouse et al. 2007).

### Sequence Divergence

Genomic variation can be quantified by the coding substitutions that have accumulated in a gene. We applied tests for signatures of positive selection using the models of codon substitution implemented in PAML (Yang 2007) to a subset of 124 genes (i.e., excluding conserved paralogs and alignments with multiple copies of one gene in one species). The majority of the hemopoiesis genes were highly conserved, except for five genes (*Ser*, *Dpp*, *ush*, *cher,* and *sgg*) involved in hemocyte differentiation. Of the 92 candidate genes that are induced upon parasitization (excluding the conserved paralogs), 23 showed signs of positive selection (table 2). Fourteen of the genes under positive selection are proteases, and three of these proteases (*CG4259*, *CG18477,* and *CG6639*), are expressed primarily in hemocytes (Irving et al. 2005). Using electronic prediction (in Pfam: Punta et al. 2012), we found that in 4 of the 14 proteases, the sequence variation led to changes in the functional domain among species (supplementary table S5, Supplementary Material online).

**Table 2:** Candidate Genes Showing Positive Selection

Recognition	Signaling	Effector	Proteases	Hemopoiesis
Hml, Corin, αPS4, TepI, TepII, TepIV	nec	yellow-f, Cyp309a1	CG11313*, CG30414*, CG4259*, CG9673*, CG12951, CG17278, CG18477, CG17572, CG31780, CG3916, CG30090, CG6639, CG9676, Jon65Aiii	Ser, Dpp, ush, cher, sgg

Note.—Sequence divergence in genes in the hemopoiesis pathway or in 92 genes that were overexpressed after parasitization. The genes showing positive selection, based on models of codon substitution, were allocated to the five immune categories as described in the main text. The first four proteases (indicated by an asterisk) show among-species differences in functional domains (supplementary fig. S5, Supplementary Material online).

Of the seven putative recognition proteins under positive selection, five are also involved in the humoral response, whereas *αPS4* is exclusive to the cellular response (Irving et al. 2005). A second recognition protein exclusive to the cellular response, *lectin-24A* (Keebaugh and Schlenke 2012), was found to be significant in the PAML analysis but not after FDR correction. Recognition proteins that were significant for positive selection share a common pattern: they were expressed later in the response against wasp attack, suggesting that they act downstream in the reaction cascade. This contrasts with recognition proteins that show high conservation, both in terms of ortholog numbers and protein-coding substitutions, such as PGRPs and GNBPs (peptidoglycan recognition proteins and gram-negative binding proteins, respectively), which are expressed early during the response and can be thus considered to be upstream in the cascade (supplementary fig. S4, Supplementary Material online).

### Lineage Specific Gains and Losses

**Fig. 3.—:**
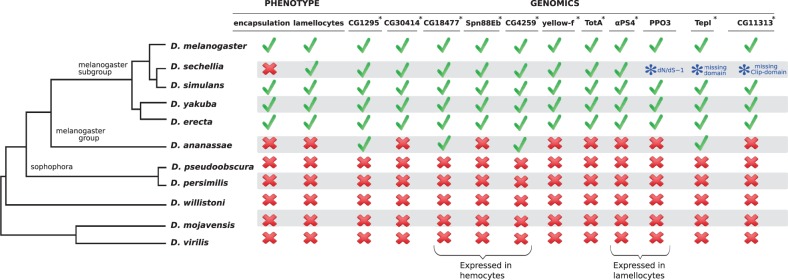
Correlations for phenotypes and lineage-restricted genes. Correlation of phenotypic characterization and the pattern of presence–absence and major genomic changes of 11 newly acquired genes between the closed interval of *melanogaster* group and subgroup. Asterisks indicates genes under positive selection.

**Fig. 4.—:**
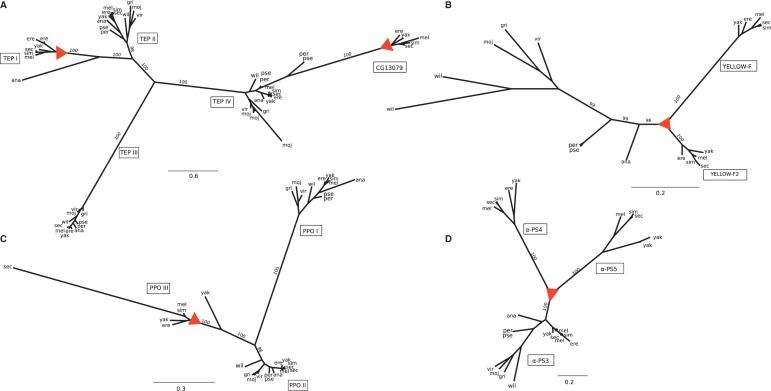
Phylogeny of orthologous groups with recent duplications. (*A*) *Tep*, (*B*) *yellow*, (*C*) *PPO,* and (*D*) *αPS*. Triangles depict expansions in the *melanogaster* group and subgroup. Bootstrap values are shown for major subgroups. The scale bar corresponds to estimated amino acid substitutions per site.

Among all the 26 LR genes, only five have a homolog outside the *melanogaster* group (table 3, supplementary table S4, Supplementary Material online), and 11 LR genes appear in the closed interval between the *melanogaster* group and subgroup, that is, the interval that contains species able to encapsulate by means of lamellocytes (fig. 3). Genes can be restricted to a certain lineage due to duplications in a specific branch, to de novo appearance, or because they have diverged from their orthologs beyond recognition (Tautz and Tomislav 2011). For four of the LR (*yellow-f*, *PPO3*, *αPS4,* and *TepI*), we established that they are recent duplications (fig. 4). For the remaining LR, additional outgroups would be necessary to detect the timing of the duplication event. Nonetheless, most genes appear to be part of large gene families, suggesting a combination of duplication and rapid accumulation of coding mutations.

**Table 3 evu012-T3:** LR Genes Associated with the Cellular Immune Response

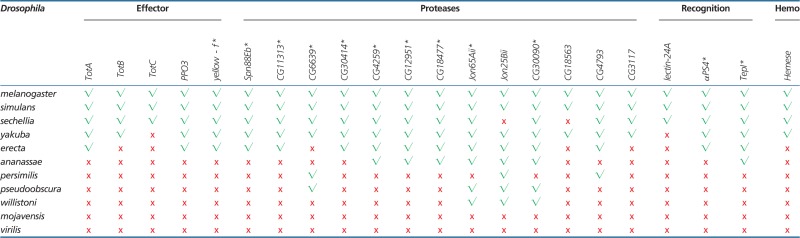

Note.—LR genes in the hemopoiesis pathway (“hemo”) or among the genes that are overexpressed after parasitization in microarray studies (Wertheim et al. 2005, Schlenke et al. 2007). The genes are allocated to five immune categories, as indicated in the main text. The presence (green check mark) or absence (red cross mark) of orthologs is indicated for the 22 genes (out of the 26 LR genes in total) that had a copy in at least three species. Only five genes have a copy outside the *melanogaster* group. Asterisk denotes genes that are also under positive selection.

Three of the LR, *TepI*, *PPO3,* and *CG11313*, showed large-scale differences in the sequence of *D. sechellia*, the only representative of the *melanogaster* subgroup unable to encapsulate. These patterns might be associated with the loss of the encapsulation trait, for example, through relaxed stabilizing selection. A detailed examination of *TepI* revealed a major deletion of four exons in *D. sechellia*, which are all present in the remaining species (supplementary fig. S3, Supplementary Material online, note that the predicted gene product did not fully correspond to our sequenced transcript). The *PPO3* gene showed a disproportionately long phylogenetic distance to the rest of species (fig. 4*C*). Pairwise estimations of substitution rates suggest a neutral substitution rate in *D. sechellia*, whereas this gene seems to be under stabilizing selection in the other species (supplementary table S2, Supplementary Material online). A closer look into the alignment of the *PPO3* protein shows that of the three domains predicted through Pfam, the first two are lost in *D. sechellia* (supplementary fig. S6, Supplementary Material online). Of the three prophenoloxidase (PPO) coding-genes in the *D. melanogaster* genome (Tang 2009), the products of *PPO1* and *PPO2* are primarily expressed in crystal cells, whereas the expression of *PPO3* is restricted to lamellocytes (Irving et al. 2005). All 11 species produced crystal cells and possessed the genes *PPO1* and *PPO2*, but only the species that produced lamellocytes possessed gene *PPO3*. Finally, *CG11313* showed a lack of clip domain in *D. sechellia*, which is present in the rest of the species of the *melanogaster* subgroup (supplementary fig. S5, Supplementary Material online). Clip is a regulatory domain that controls the proteinase action during activation and regulation of protease cascades (Piao et al. 2005). Although the specific immune function has not yet been described for this gene, its high rate of amino acid substitutions suggests directional selection. Possibly, the loss of the clip domain in *D. sechellia* is accompanied by a new function rather than loss of function.

### Comparative Expression of *TepI*, *PPO3*, and *IM1*

**Fig. 5.—:**
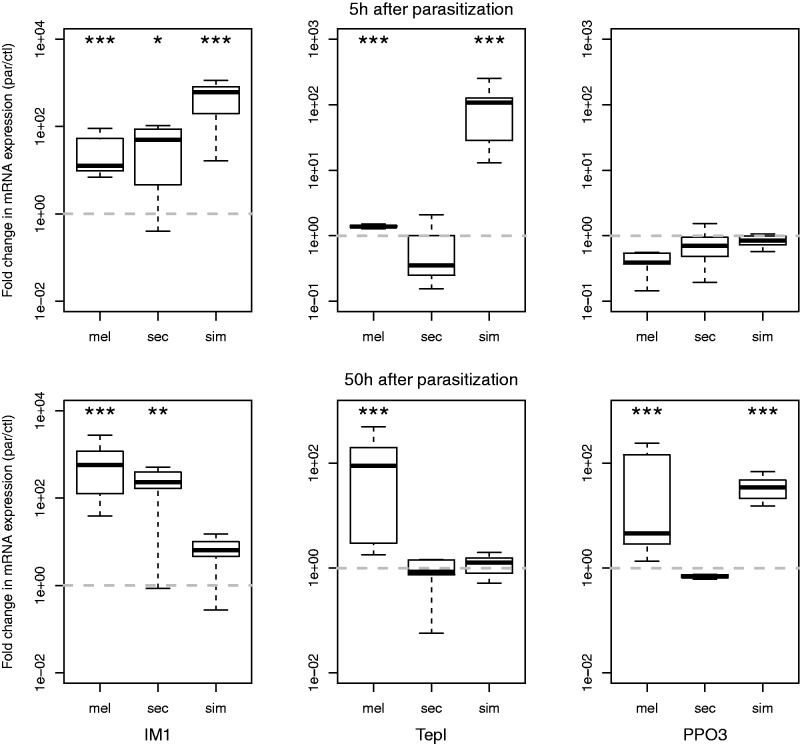
Fold-changes in expression of *IM1*, *TepI,* and *PPO3* after parasitization. The ratio between parasitized and control expression levels is calculated and normalized by two reference genes. Boxplots depict the distribution of the replicates and the error estimated through permutation. The dotted gray line describes the value for which the ratio is one (i.e., no induced expression). Significance level: 0.05*, 0.005**, 0.001***.

To 1) test for species differences in the (level of) activation of the well-established signal transduction pathways in the immune response against parasitoids (Toll, Jak/Stat, and Prophenoloxidase) and 2) gain insight into the relation between the substantial genomic changes in *D. sechellia* and this activation, we performed RT-qPCR assays. We compared the fold changes in expression of *TepI*, *PPO3,* and *IM1* for larvae at two time points (5 and 50 h) after parasitization, among the three sister species, *D. melanogaster*, *D. simulans,* and *D. sechellia*. *TepI* and *PPO3* are the diverged targets of the Jak/Stat pathway and Phenoloxidase cascade, respectively, whereas *IM1* is a conserved target of the Toll pathway (fig. 5). Apart from its role as indicator for the activation of the Toll pathway, *IM1* could also be considered a more general indicator for immune activation, as it is induced in response to a variety of immune challenges (Kraaijeveld and Wertheim 2009).

Five hours after parasitization, *IM1* was induced in larvae of all three species, indicating that all species activated the Toll pathway and responded to the immune challenge. *TepI* was strongly induced 5 h after parasitization in *D. simulans,* and in *D. melanogaster* at very low levels at 5 h and strongly at 50 h, indicating that *D. melanogaster* and *D. simulans* species activated the Jak/Stat pathway, but *D. simulans* did so faster. In *D. sechellia, TepI* was expressed only at 50 h, but at similar levels in control and parasitized groups. *PPO3* was not differentially expressed 5 h after parasitization in any *Drosophila* species but was up-regulated at 50 h after attack in *D. melanogaster* and *D. simulans*. Interestingly, no expression of *PPO3* was found in *D. sechellia*, which is consistent with a loss of function for *PPO3*.

## Discussion

From the species we tested, those outside the *melanogaster* subgroup were unable to encapsulate eggs of the parasitoids *A. tabida* or *A. citri* and also did not produce lamellocytes, a specialized type of blood cell important for the encapsulation process. Importantly, the production of lamellocytes and the presence versus absence of encapsulation ability among the 11 surveyed *Drosophila* species is not specific to the *Asobara* parasitoids, but most likely representative for parasitoid wasps in general, as evidenced by very similar patterns among *Drosophila* exposed to the distantly related *Leptopilina* parasitoids (Schlenke et al. 2007). Lamellocytes were previously found to be lacking in some *Drosophila* species that did not mount immune responses against parasitoid wasps (Havard et al. 2009), which was considered a loss of the trait (Eslin and Doury, 2006). Conversely, our study combined with data on other species (Schlenke et al. 2007) indicates that lamellocyte-mediated encapsulation is not a common trait, shared among all *Drosophila* species, but appears to be restricted to only a subset of species. Older references reported encapsulation ability outside the *melanogaster* group, in *D. algonquin* from the *obscura* group (Nappi 1975), and in a distantly related species of the subgenus *Dorsilopha*, *D. busckii* (Streams 1968), but it appears that the mechanisms are not likely to be the same. In some of the species of the *obscura* group that lack lamellocytes, including the aforementioned *D. algonquin*, the encapsulation process is mediated by a different type of hemocyte, the pseudopodocytes (Havard et al. 2012). Although hemocytes have traditionally been identified through morphology, the use of molecular markers is helping to resolve some of the controversies from the morphological classification of the different hemocyte types. We found that some of the commonly used markers for lamellocytes (*αPS4* and *Hemese* [Kurucz et al. 2007, Havard et al. 2012]) are genes restricted to the clade able to produce lamellocytes. This could indicate that blood cells involved in encapsulation in more distantly related species are of a different type as was also found in the *obscura* group and might explain why no labeling is observed in species of this group when using the available antibodies built against *D. melanogaster* hemocytes (Havard et al. 2012).

Outside *Drosophila*, encapsulation has also been reported in the orders Lepidoptera and Orthoptera (among others) (Strand and Pech 1995). Although less is known about encapsulation in Orthoptera, the encapsulation process in Lepidoptera is one of the functions of granulocytes and plasmatocytes, which do not seem to be the equivalent of lamellocytes in *Drosophila* (Ribeiro and Brehelin 2006). There is also much variation for mechanisms underlying encapsulation in Dipteran species outside the *Drosophila* genus. In mosquitoes, encapsulation occurs by a sheath of melanin in the absence of a multicellular layer, which is referred to as humoral encapsulation (Vey 1993). The clear division of function between phagocytic and adhesive cells has not been found in mosquitoes (Castillo et al. 2006). In house flies (*Musca*), nematodes are also encapsulated by a sheath of melanin, which is then covered by a syncytial mass of host hemocytes, probably of oenocytoid origin (Nappi and Stoffolano 1971). This variety in the blood cell types among insects reflects the plastic nature of the hemolymphatic tissue and makes it difficult to establish the homology of the mechanism. To fully understand whether lamellocyte-mediated encapsulation represents an acquired novel trait or whether it has been lost multiple times during evolution requires the investigation of additional species, additional strains for both host and parasitoid species, and rigorous phylogenetic comparisons of the type of blood cells, encapsulation process, and genes involved.

In this study, we focused on the evolutionary genomics underlying the striking phenotypic variation in *Drosophila* and investigated the gain/loss and diversification of genes that underlie lamellocyte differentiation and melanotic encapsulation. Using a comparative genomics approach, we show that the presence of lamellocytes and encapsulation ability is associated with the evolution of various novel genes and rapid divergence in (new) protein-coding genes (fig. 2). We followed up on genes associated with hemopoieses and genome-wide expression studies after parasitoid attack to identify genes putatively involved in the melanotic encapsulation response. Although we do not claim complete inclusion of all relevant genes for encapsulation ability, and we are likely to miss noncoding regions or genes with small effects (i.e., genes that were not significant in the expression study or whose phenotypic effects are not yet identified or imperceptible), we obtained a more comprehensive list of candidate genes that reflects the process of differentiation and proliferation of blood cells upon parasitization, as well as other aspects of the encapsulation defenses. Our comparative analyses revealed that, except for *Hemese*, all hemopoiesis-associated genes are highly conserved and present in all species independently of their resistance. Only 5 of the 35 hemopoietic genes showed signs of positive selection, and these five are associated with the process of hemocyte differentiation. This relatively low proportion is not too surprising, considering that genes involved in hemocyte proliferation and differentiation are also implicated in a variety of other biological and developmental processes, and their evolution is therefore likely to be highly constrained. In contrast, of the genes differentially expressed after parasitoid attack, 25 were novel genes, of which only five have homologs outside the *melanogaster* group, and 23 genes were significant for positive selection, mostly proteases and recognition genes.

In an attempt to identify candidate genes underlying the evolution of parasitoid resistance, we specifically focused on the novel genes. Although it would be tempting to hypothesize that the acquisition of the only LR gene in the hemopoiesis pathway, *Hemese*, is responsible for the origin of lamellocytes, this may be premature. *Hemese* is expressed in all hemocytes, whereas inhibition of its expression by RNAi enhances both the proliferation of hemocytes and the production of lamellocytes after parasitoid attacks (Kurucz et al. 2003). It, therefore, appears that *Hemese* functions as a negative regulator of lamellocyte differentiation, fine tuning the activation and recruitment of hemocytes, rather than in initiating lamellocytes differentiation (Kurucz et al. 2003). The other genes known for lamellocyte differentiation are common to all 11 species, indicating that existing (hemopoiesis) genes have been co-opted for the acquisition/evolution of a new type of hemocytes. Of the remaining 25 novel genes, 13 were significant for positive selection (supplementary table S4, Supplementary Material online), and of these, seven (*TepI*, *αPS4*, *lectin-24A*, *CG4259*, *CG18477*, *Spn88Eb7,* and *PPO3*) are mainly or exclusively expressed in hemocytes or lamellocytes (Irving et al. 2005). Four of the novel genes were derived from recent duplications, and most others also appear to be (new) members of large gene families. The combined patterns suggests neofunctionalization of duplicated genes, where they evolved new functions associated with lamellocyte differentiation and melanotic encapsulation. The signature of positive selection in the duplicated genes may reflect the neofunctionalization process itself, where the sequences evolve to optimize their new function, whereas it could also reflect the strong selection pressures that may occur in host–parasite coevolution. Although detailed functional studies of these genes are required to confirm their precise role in the cellular immune response (currently under research), we hypothesize that they may be instrumental in the evolution of parasitoid resistance in the *Drosophila* lineage.

Of the novel genes, three show considerable changes exclusive to *D. sechellia*, which secondarily lost resistance. Three genes (*TepI, PPO3,* and *CG11313*) show a loss of putative functional domains in *D. sechellia* (supplementary figs. S3, S5, and S6, Supplementary Material online). Our expression study indicated that *TepI* was expressed but not significantly induced after parasitization in *D. sechellia*, thus it is not clear the degree to which *TepI* retained some functionality in this species. *PPO3* seemed to accumulate coding mutations at a neutral rate in *D. sechellia,* whereas the gene is under strongly purifying selection in the other *Drosophila* species. These changes suggest a release of the selection pressure for this gene, and the complete lack of expression of this gene in *D. sechellia* strongly supports that its function is lost. Especially the three genes that show a loss of a functional domain for protein interactions in *D. sechellia* could provide strong candidates for genes involved in the secondary loss of the encapsulation ability in this species, although these molecular signatures could also reflect a relaxation from balancing selection. Fast changes and loss of genes in *D. sechellia* have been shown to occur during its resource specialization on “noni” fruit (McBride 2011). An interesting question is whether the lack of resistance against parasitoid wasps is also a consequence of the specialization to this resource that is toxic to other *Drosophila* species. Our preliminary results indicate that this fruit indeed is toxic to parasitoids too, which would imply that *D. sechellia* may have lost its immunological resistance to parasitoids, because it is living in an enemy-free niche.

Previous genomic studies argued that divergence in genes involved in antagonistic host–parasite interactions should happen more often in: 1) immune pathways that are targeted and suppressed by parasites (which is apparently the case for IMD and RNAi) and 2) receptors that are in direct contact with the pathogens (Obbard et al. 2009). Parasitoid counter-defense strategies include the injection of immunosuppressive virulence genes coming from DNA viruses (Bitra et al. 2012) and the production of RhoGAP toxins by the parasitoids that induce changes in morphology and adhesion properties of host hemocytes (Colinet et al. 2007). Unfortunately, the immune suppressive effects of parasitoids remain much less understood than the immune response of the host, and even for the latter, the molecular mechanisms for parasitoid recognition are not known. The rapid evolution of certain immune genes within the recognition class in our analyses suggests that the position of genes in the reaction cascade is also important for their evolutionary dynamics. Of the 15 recognition genes in our candidate list, six genes were under positive selection. All these genes are expressed at later stages during the immune response, suggesting that they act downstream in the reaction cascade, for example, by directing the cellular response toward the foreign body. In contrast, four recognition genes with high conservation in terms of both number of orthologs and amino acid sequence (e.g., PGRPs) are upregulated immediately after the immune challenge (fig. 2*B*, supplementary fig. S4, Supplementary Material online), suggesting they act upstream, triggering the reaction cascade. Unfortunately, for the remaining five recognition genes, no expression profile was available for early time points. The divergent evolutionary patterns for the upstream and downstream recognition genes could be the consequence of different constraints. The effects of genes that act upstream is amplified along the cascade, and changes in their protein-coding sequence can have profound consequences on the triggered response (Sackton et al. 2010). The high conservation both in ortholog number and coding sequences could thus be the consequence of selection acting to preserve a mechanism that evolved even before the diversification of insects. Other receptor genes that act downstream in the immune response (*TepI*, *lectin-24A*, and *αPS4*), would be less constrained by this amplification effect, having thus more potential to change.

Our study on the cellular immune response complements the insights that previous genomic studies on the humoral and RNAi immune responses have established in *Drosophila* (Sackton et al. 2007; Obbard et al. 2009). Consistent with these studies, we find that most of the protein-coding genes involved in the immune response show high conservation, both in terms of number of orthologs and coding substitutions. Similarly, we find that effector genes diversify mainly through gene duplication. Different to previous studies, we combined a comprehensive list of candidate genes associated with hemopoieses and the response to parasitoid attack. We found that an important number of the up-regulated genes are fast evolving genes or novel genes, whereas most of the hemopoietic genes are highly conserved. Our study also highlights the importance of proteases in the evolution of the cellular immune response. Proteases were not only the largest class of proteins (45) but also the one containing most of the duplicated genes and genes under positive selection (17 and 14, respectively). At present, proteases appear to be fundamental mediators in regulatory processes (Jang et al. 2008). Our finding of both high rates of duplication and protein-coding substitution indicates that once a new protease copy arises, it can diversify to generate new outcomes of existing pathways. Such rapid change suggests that proteases are “easily” recruited in existing pathways, and in the case of the cellular immune response, this rapid change may play a pivotal role in coordinating differentiation and movement of cells on which the cellular response relies.

An important question that remains to be explored is under what circumstances the ability to encapsulate evolved in a certain group and why it was lost in some species. The molecular mechanisms for the emergence of novel traits and, more dramatically, the loss of traits that were thought to be essential is currently a hot topic (Johnson and Tsutsui 2006; Rebeiz et al. 2011; Star et al. 2011). These studies have profited enormously from genomics approaches, because only through this whole-genome approach, genes are studied in the genomic context where they evolved.

In conclusion, through a combination of phenotypic and genomic characterizations we provide an important step toward understanding the evolution of the cellular resistance against parasitoids in *Drosophila* species. We highlight specific protein-coding genes that are likely to be important in the acquisition and subsequent loss of this trait, bridging the gap between phenotype and genotype. Understanding the detailed processes underlying the evolution of the encapsulation ability in *Drosophila* may also give insights into the evolution of immune traits in general. *Drosophila* has been long recognized as an excellent model organism for revealing the molecular mechanisms of innate immunity and hemopoiesis also in vertebrates (Williams 2007). Interestingly, the immune response of vertebrates relies largely on a variety of differentiated blood cells. We showed that a combination of co-option and neofunctionalization is likely to have contributed to the acquiring of the new immunity component in the cellular immune response and that particular gene families (serine-type proteases, Tep and lectins) could be of special interest for the processes of hemocyte differentiation, proliferation, and activation. It would be of great interest to study the role of these gene families in the evolution of the large versatility in blood cells in vertebrates and invertebrates.

## Supplementary Material

Supplementary figures S1–S7 and tables S1–S5 are available at *Genome Biology and Evolution* online (http://www.gbe.oxfordjournals.org/).[Table evu012-T3]

Supplementary Data
