# Supplementary material for: Evolution of a Cellular Immune Response in Drosophila: A Phenotypic and Genomic Comparative Analysis
Source: Genome Biol Evol. 2014 Jan 18;6(2):273–89. doi: 10.1093/gbe/evu012 (PMC3942026; doi:10.1093/gbe/evu012)
Supplement: Supplementary Data [file supp_6_2_273__index.html]

Evolution of a Cellular Immune Response in Drosophila: A Phenotypic and Genomic Comparative Analysis — Supplementary Data 

# Evolution of a Cellular Immune Response in *Drosophila*: A Phenotypic and Genomic Comparative Analysis

## Supplementary Data

files

**Files in this Data Supplement:**

- Supplementary Data - pdf file
