# Supplementary material for: Evolution of a Cellular Immune Response in Drosophila: A Phenotypic and Genomic Comparative Analysis
Source: Genome Biol Evol. 2014 Jan 18;6(2):273–89. doi: 10.1093/gbe/evu012 (PMC3942026; doi:10.1093/gbe/evu012)
Supplement: Supplementary Data [file supp_evu012_SalazarSupport.pdf]

Genome changes associated with the evolution of the cellular immune response in *Drosophila* species: Supplementary Material

Laura Salazar-Jaramillo[1,*], Angeliki Paspati[1], Louis van de Zande[1], Cornelis Joseph Vermeulen[1], Tanja Schwander[2], Bregje Wertheim[1,*]

1 Evolutionary Genetics/Center for Ecological and Evolutionary Studies, Groningen University, Groningen, The Netherlands

2 Department of Ecology and Evolution, Biophore, University of Lausanne, Lausanne, Switzerland

∗ **E-mail: b.wertheim@rug.nl, lauraalazar@gmail.com**

Supplementary Figures

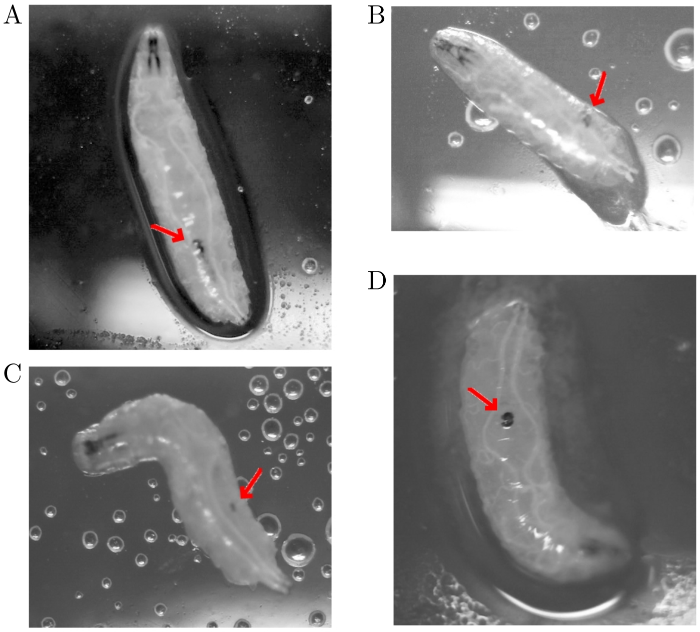

Figure S1. Examples of melanization in larvae. A: *D. annanassae* after 2h, B: *D. pseudoobscura* after 4h, C: *D. simulans* after 2h , D: *D. virilis* after 2h

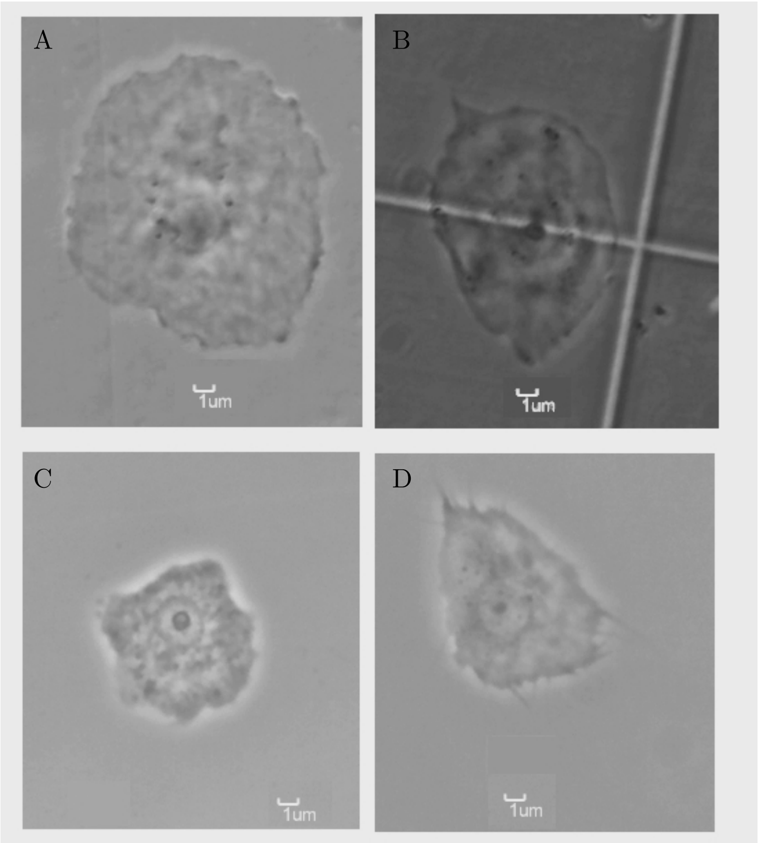

Figure S2. Example of lamellocytes in parasitized 3rd instar larvae. A: *D. melanogaster*, B: *D. simulans*, C,D: *D. willistoni*. The morphology of the cells in *D. willistoni* share some features with lamellocytes, such as a flat shape and the presence of pseudopodia, however their size is not as big as a typical lamellocyte and a halo of light suggests that they are not completely flat

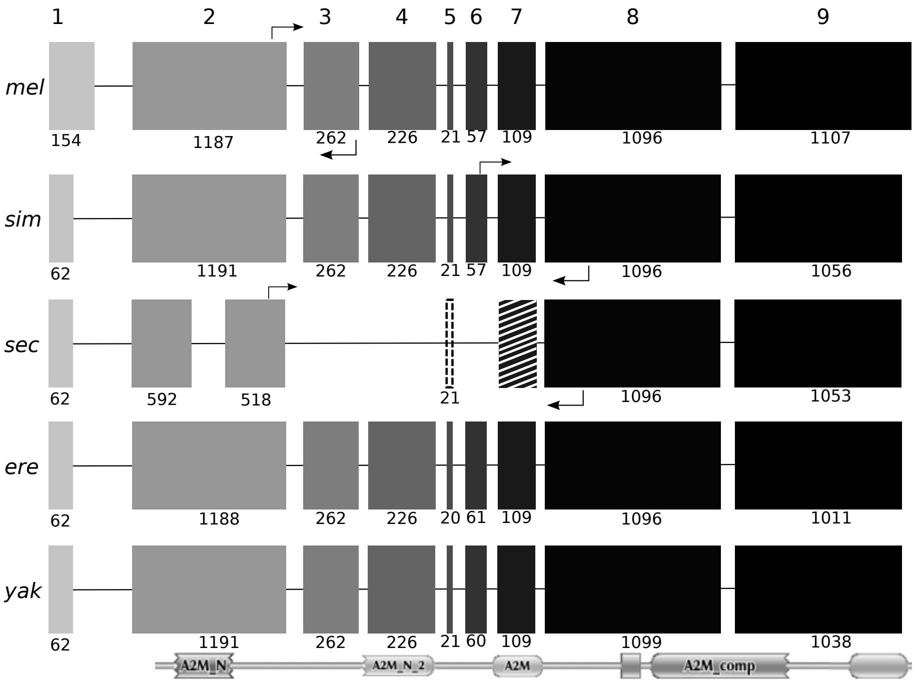

Figure S3. Scheme of TepI exon-intron alignment Exons are represented by squares and their respective approximate functional domains from Pfam is shown at the bottom (A2M:α2Macroglobulin family, N: N terminal region, comp: complement component, recep: receptor, square: thiol-ester bond-forming region). Arrows depict the position of the primers for the species, for which expression was measured (*D. melanogaster, D. simulans, D.sechellia*). We sequenced the qPCR product of *D. sechellia* because the amplicon size was considerably larger than predicted, and found two differences with respect to the prediction: predicted exon 5 is not found in the mRNA sequence (non-filled square), and a predicted intron in position 7 was found in the sequenced mRNA (stripped square)

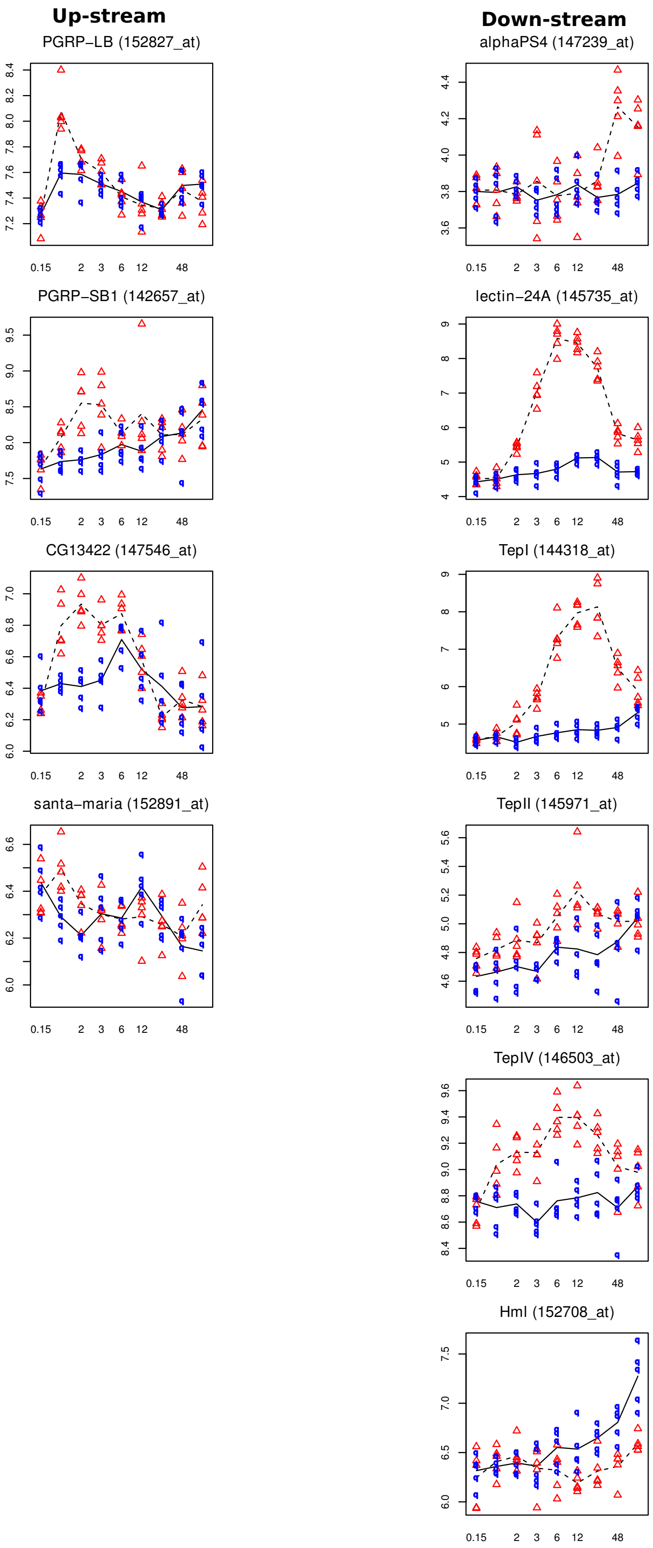

Figure S4. Two types of receptors Receptors that are up-stream the signalling pathways express earlier (i.e expression peak is in the first 3 hours after parastization), whereas activated receptors down-stream the signalling pathways express later (i.e expression peak after 5 hours). Although our list of candidate genes included data from both studies, [4] and [3], the expression pattern is based only on [4], because in [3] there are no measurements earlier than 5 hours. Reproduced with permission from [4].

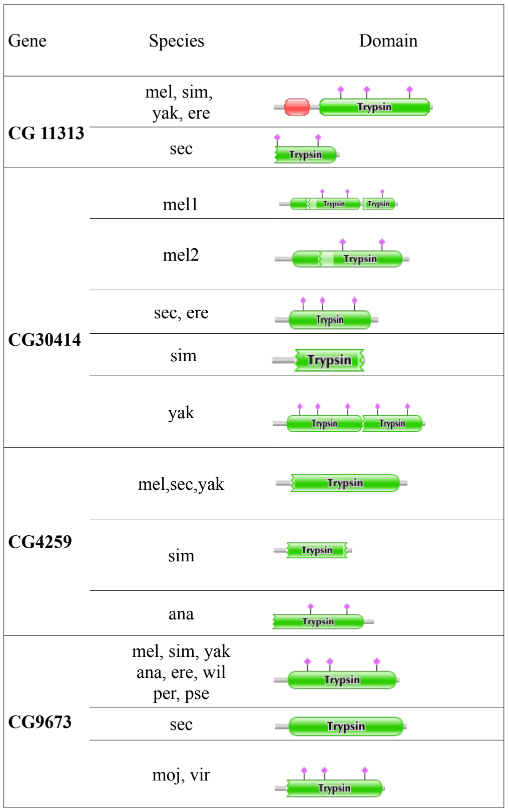

Figure S5. Serine proteases under positive selection with differences in functional domains
Green: trypsin, red: clip domain. Each pink stick correspond to one residue of the catalytic triad: histidine, cystein and serine

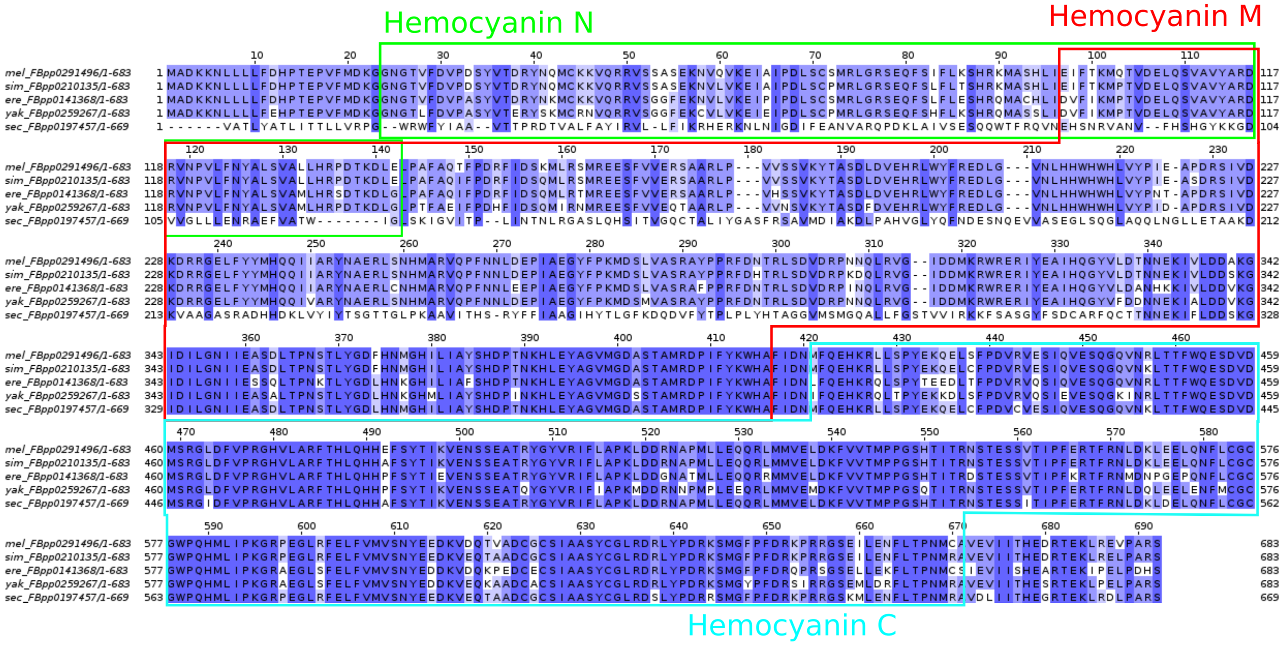

Figure S6. Multiple Alignment of PPO3 The functional domains predicted by PFAM are highlighted

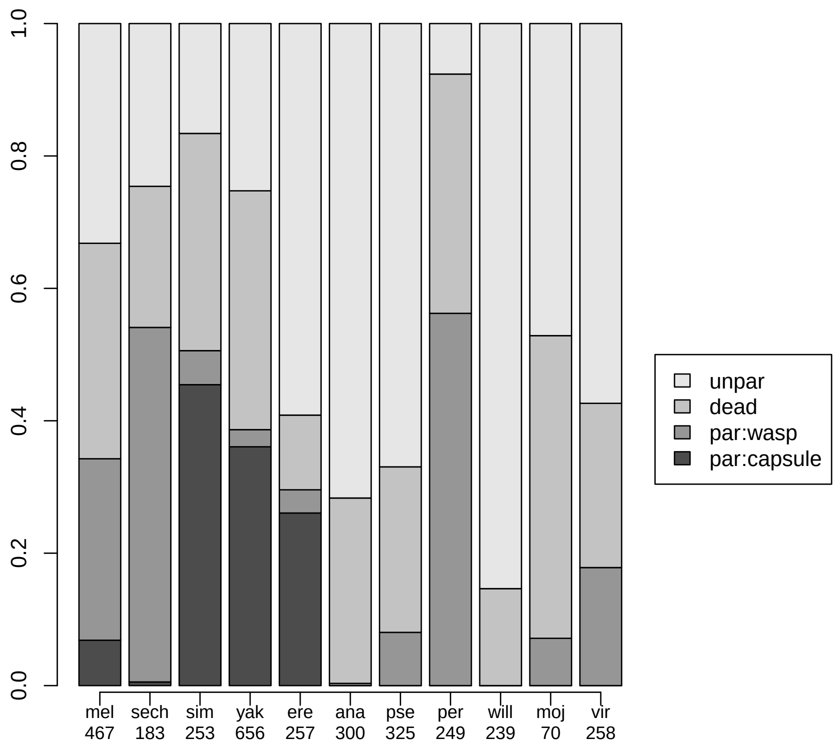

Figure S7. Summary of the parasitization experiment The total of individuals tested are pooled for all replicates and stages (dissected larvae and adults). In the x axis are the species with the total number of individuals tested. The y axis shows the outcome for each individual as proportions of: parasitized individuals that formed a capsule (par:capsule), parasitized individuals that led to wasp emergence (par:wasp), individuals that died (dead) estimated from the difference between number of pupae and emerging adults (flies or wasps), and unparasitized individuals (unpar) where no signs of capsules were found.

Supplementary Tables

Table S1. Insect strains information

**Species**	**abbreviation**	**Reference**	**Location**	**Date of collection**
D. ananassae	ana	140240371.13	Hawaii (USA)	1945
D. erecta	ere	140210224.01	Unknown	Unknown
D. melanogaster	mel	140210231.36	Unknown	Unknown
D. mojavensis	moj	150811352.22	California (USA)	2002
D. persimilis	per	140110111.49	California (USA)	1997
D. pseudoobscura	pse	1401 − 0121.94	Colorado (USA)	1996
D. sechellia	sec	14021 − 0248.25	Cousin Island (Seychelles)	1980
D. virilis	vir	150101051.87	Unknown	Unknown
D. willistoni	will	140300811.24	Guadaloupe Island (France)	Unknown
D. yakuba	yak	140210261.01	Liberia	1983
A. tabida			Sospel (France)	1994
A. citri			Ivory Coast	1995

Table S2. Pair-wise estimation of dN and dS for *PPO3*

sp1	sp2	S	N	t	kappa	omega	dN +- SE	dS +- SE
sim	mel	298.5	1681.5	0.1380	1.6559	0.0489	0.0117 +- 0.0027	0.2393 +- 0.0351
ere	mel	320.2	1659.8	0.4208	1.6559	0.0928	0.0543 +- 0.0059	0.5857 +- 0.0767
ere	sim	310.6	1669.4	0.4058	1.6559	0.0979	0.0553 +- 0.0059	0.5650 +- 0.0763
yak	mel	309.0	1671.0	0.4685	1.6559	0.0923	0.0616 +- 0.0063	0.6675 +- 0.0931
yak	sim	299.1	1680.9	0.4380	1.6559	0.0952	0.0599 +- 0.0062	0.6295 +- 0.0864
yak	ere	320.3	1659.7	0.3629	1.6559	0.1827	0.0702 +- 0.0067	0.3841 +- 0.0499
sec	mel	331.2	1648.8	1.2615	1.6559	0.4032	0.3371 +- 0.0172	0.8360 +- 0.0941
sec	sim	321.7	1658.3	1.1933	1.6559	0.4505	0.3320 +- 0.0170	0.7369 +- 0.0802
sec	ere	342.4	1637.6	1.6761	1.6559	0.2710	0.3813 +- 0.0189	1.4070 +- 0.2733
sec	yak	331.6	1648.4	1.7598	1.6559	0.2202	0.3682 +- 0.0183	1.6724 +- 0.4014

Rates estimated between two species (sp1 and sp2) by the method of Yang and Nielsen (2000) [5] implemented in the program yn00 of PAML: number of synonymous (S) and non-synonymous (N) sites in a sequence, time (t), numbers of synonymous (dS) and non-synonymous (dN) substitutions per site, omega (dN/dS), transition/transversion ratio (kappa).

Table S3. Genomic characterization

	Name	CG	Fbgn	Fbpp	Immune	mel	sim	sec	yak	ere	ana	per	pse	wil	moj	vir	gri	Homology	Sackton	paralog_to	M7vsM8	FDR
1	AttA	CG10146	FBgn0012042	FBpp0086567	effector	2	2	1	2	2	1	3	2	1	1	2	1	par	par	AttB		
2	AttB	CG18372	FBgn0041581	FBpp0086568	effector	2	2	1	1	2	1	3	3	1	1	2	1	par	par	AttA		
3	AttD	CG7629	FBgn0038530	FBpp0082908	effector	1	1	1	1	1	1	1	1	1	1	1	1	sco	sco		1.00	0.81
4	proPO59	CG42640	FBgn0261363	FBpp0291496	effector	1	1	1	2	1	0	0	0	0	0	0	0	rec_par	par	PPO1_2	1.00	0.81
5	cact	CG5848	FBgn0000250	FBpp0080402	signalling	1	1	1	1	1	1	1	1	1	1	1	1	sco	sco		0.49	0.81
6	CecC	CG1373	FBgn0000279	FBpp0084980	effector	2	2	2	1	2	1	0	4	2	1	4	4	par	par	CecA2		
7	CG11313	CG11313	FBgn0039798	FBpp0289924	protease	1	1	1	1	3	0	0	0	0	0	0	0	lr			0.00	0.00
8	CG11912	CG11912	FBgn0031248	FBpp0077758	protease	1	1	1	1	1	1	1	1	1	2	1	1	sco			0.66	0.81
9	CG12951	CG12951	FBgn0037677	FBpp0081536	protease	1	1	1	1	1	1	0	0	0	0	0	0	lr			0.02	0.07
10	et	CG14225	FBgn0031055	FBpp0074526	signalling	1	1	1	1	1	1	1	1	0	1	1	1	sco	lr		0.25	0.51
11	CG15065	CG15065	FBgn0040734	FBpp0085848	effector	1	1	1	2	1	1	2	1	2	1	1	3	par			1.00	0.81
12	CG16704	CG16704	FBgn0031558	FBpp0077239	protease	1	1	1	1	1	2	2	2	1	3	1	4	par			1.00	0.81
13	CG16712	CG16712	FBgn0031561	FBpp0077235	protease	1	1	1	1	1	1	1	1	0	1	1	1	sco			0.97	0.81
14	CG16713	CG16713	FBgn0031560	FBpp0077236	protease	1	1	1	1	1	1	1	1	1	2	1	4	par			1.00	0.81
15	CG17278	CG17278	FBgn0046763	FBpp0083449	protease	1	1	1	1	1	1	1	1	1	1	1	1	sco			0.01	0.04
16	CG17475	CG17475	FBgn0038481	FBpp0082858	protease	1	1	1	1	1	1	1	1	1	1	1	1	sco			1.00	0.81
17	CG17477	CG17477	FBgn0038479	FBpp0082859	protease	1	1	1	1	1	1	1	1	1	1	1	1	sco			0.99	0.81
18	CG17572	CG17572	FBgn0032753	FBpp0080715	protease	1	1	1	1	1	1	1	1	1	1	1	1	sco			0.00	0.00
19	CG18477	CG18477	FBgn0028864	FBpp0080315	protease	2	1	1	1	1	2	0	0	0	0	0	0	lr		CG31780	0.00	0.00
20	CG18478	CG18478	FBgn0028517	FBpp0080316	protease	2	0	0	1	0	0	0	0	0	0	0	0	lr		CG31827		
21	CG18563	CG18563	FBgn0032639	FBpp0290124	protease	1	1	0	1	0	0	0	0	0	0	0	0	lr			0.10	0.28
22	CG2736	CG2736	FBgn0035090	FBpp0072310	recognition	1	1	1	1	1	1	1	1	1	1	1	1	sco			0.20	0.45
23	CG30090	CG30090	FBgn0050090	FBpp0086417	protease	1	1	1	1	1	0	1	1	0	0	0	0	lr			0.00	0.00
24	CG30371	CG30371	FBgn0050371	FBpp0087874	protease	1	1	1	1	1	1	1	1	1	1	1	1	sco			1.00	0.81
25	CG30414	CG30414	FBgn0050414	FBpp0071941	protease	1	1	1	1	1	0	0	0	0	0	0	0	lr			0.00	0.00
26	CG3117	CG3117	FBgn0031471	FBpp0289585	protease	1	1	1	1	0	0	0	0	0	0	0	0	lr			0.65	0.81
27	CG31266	CG31266	FBgn0051266	FBpp0289337	protease	1	1	1	1	1	1	1	1	1	1	1	1	sco			1.00	0.81
28	CG31269	CG31269	FBgn0051269	FBpp0112313	protease	1	1	1	1	1	1	1	1	1	1	1	1	sco			1.00	0.81
29	CG31780	CG31780	FBgn0051780	FBpp0080317	protease	2	1	1	1	1	2	0	0	0	0	0	0	lr		CG18477	0.00	0.00
30	CG31827	CG31827	FBgn0051827	FBpp0080318	protease	2	0	0	1	0	0	0	0	0	0	0	0	lr		CG18478		
31	CG32374	CG32374	FBgn0052374	FBpp0076539	protease	2	1	1	2	1	2	1	1	1	0	1	1	par		CG32376		
32	CG32376	CG32376	FBgn0052376	FBpp0076538	protease	2	1	1	2	1	2	1	1	1	0	1	1	par		CG32374		
33	CG32483	CG32483	FBgn0052483	FBpp0072504	protease	2	2	1	2	2	1	2	2	2	3	2	2	par		CG3344		
34	CG33127	CG33127	FBgn0053127	FBpp0077712	protease	1	0	0	0	0	0	0	1	0	0	0	0	lr			0.12	0.31
35	CG3344	CG3344	FBgn0035154	FBpp0072505	protease	2	2	1	2	2	1	2	2	2	3	2	2	par		CG32483		
36	CG3505	CG3505	FBgn0038250	FBpp0082425	protease	1	1	1	1	1	1	1	1	1	1	1	1	sco			0.49	0.81
37	CG3916	CG3916	FBgn0038003	FBpp0082032	protease	1	1	1	1	1	1	1	1	1	1	1	1	sco			0.01	0.04
38	CG4053	CG4053	FBgn0038482	FBpp0292474	protease	1	1	1	1	1	1	1	1	1	1	1	1	sco			1.00	0.81
39	CG4259	CG4259	FBgn0031389	FBpp0077526	protease	1	1	1	1	1	1	0	0	0	0	0	0	lr			0.00	0.00
40	CG4653	CG4653	FBgn0030776	FBpp0074095	protease	1	1	1	1	1	1	1	1	1	1	1	1	sco			0.97	0.81
41	CG4793	CG4793	FBgn0028514	FBpp0289839	protease	1	1	1	1	1	0	1	1	0	0	0	0	lr			0.32	0.62
42	CG5246	CG5246	FBgn0038484	FBpp0082846	protease	1	1	1	1	1	1	1	1	1	1	1	1	sco			1.00	0.81
43	CG6041	CG6041	FBgn0029826	FBpp0070826	protease	1	1	1	1	1	1	1	1	1	1	1	1	sco			1.00	0.81
44	CG6639	CG6639	FBgn0032638	FBpp0080557	protease	1	1	1	1	0	0	1	2	0	0	0	0	lr			0.01	0.04
45	Spn88Eb	CG6687	FBgn0038299	FBpp0082594	protease	1	1	1	1	1	0	0	0	0	0	0	0	lr			0.00	0.00
46	CG9240	CG9240	FBgn0030669	FBpp0073876	protease	1	1	1	1	1	1	1	1	1	1	1	1	sco			0.07	0.21
47	CG9673	CG9673	FBgn0030775	FBpp0074136	protease	1	1	1	1	1	1	1	1	1	1	1	1	sco			0.02	0.07
48	CG9733	CG9733	FBgn0039759	FBpp0085000	protease	1	1	1	1	1	1	1	1	1	1	1	1	sco			1.00	0.81
49	Corin	CG2105	FBgn0033192	FBpp0087983	recognition	1	1	1	1	1	1	1	1	1	1	1	1	sco	sco		0.00	0.00
50	Cyp12a4	CG6042	FBgn0038681	FBpp0083145	effector	3	3	3	3	3	4	5	6	5	3	3	4	par		Cyp12a5_CG13604		
51	Cyp12a5	CG11821	FBgn0038680	FBpp0083144	effector	3	3	3	3	3	4	5	6	5	3	3	4	par		Cyp12a4_CG13604		
52	Cyp12e1	CG14680	FBgn0037817	FBpp0290498	effector	1	1	1	1	1	1	1	1	2	1	1	1	sco			0.66	0.81
53	Cyp309a1	CG9964	FBgn0031432	FBpp0077429	effector	1	1	1	1	1	1	1	1	1	1	1	1	sco			0.00	0.00
54	Cyp4e3	CG4105	FBgn0015035	FBpp0079515	effector	3	3	3	3	2	3	4	4	2	1	2	1	par		Cyp4e2_Cyp4e1		
55	Cyp6a17	CG10241	FBgn0015714	FBpp0086581	effector	2	2	2	2	1	3	2	2	3	2	1	1	par		Cyp6a23		
56	Cyp9c1	CG3616	FBgn0015040	FBpp0072228	effector	1	1	1	1	1	1	1	1	1	1	1	1	sco			1.00	0.81
57	Cyp9f2	CG11466	FBgn0038037	FBpp0082070	effector	1	1	1	1	1	1	1	1	1	1	2	1	sco			0.83	0.81
58	Dhpr	CG4665	FBgn0035964	FBpp0076258	effector	1	1	1	1	1	1	1	1	1	1	1	1	sco			1.00	0.81
59	dome	CG14226	FBgn0043903	FBpp0074525	signalling	1	1	1	1	1	1	1	1	1	1	1	1	sco	sco		1.00	0.81
60	dl	CG6667	FBgn0260632	FBpp0080558	signalling	1	1	1	1	1	1	1	1	1	1	1	1	sco	sco		0.21	0.46
61	emb	CG13387	FBgn0020497	FBpp0079278	signalling	1	1	1	1	1	1	1	1	1	1	1	1	sco	sco		1.00	0.81
62	Fmo-2	CG3174	FBgn0033079	FBpp0085481	effector	1	1	1	1	1	1	1	1	1	1	1	1	sco			1.00	0.81
63	grass	CG5896	FBgn0039494	FBpp0084481	signalling	1	1	1	1	1	1	1	1	1	1	1	2	sco	sco		0.69	0.81
64	GNBP3	CG5008	FBgn0040321	FBpp0076237	recognition	1	1	1	1	1	1	1	1	1	1	1	1	sco	sco		1.00	0.81
65	CG13422	CG13422	FBgn0034511	FBpp0085532	recognition	1	1	1	1	1	1	1	1	1	1	1	1	sco	par		1.00	0.81
66	Hml	CG7002	FBgn0029167	FBpp0075495	recognition	1	1	1	1	1	1	2	2	4	1	1	1	sco	sco		0.00	0.00
67	hop	CG1594	FBgn0004864	FBpp0073313	signalling	1	1	1	1	1	1	1	1	2	1	1	1	sco	par		0.97	0.81

	Name	CG	Fbgn	Fbpp	Immune	mel	sim	sec	yak	ere	ana	per	pse	wil	moj	vir	gri	Homology	Sackton	paralog_to	M7vsM8	FDR
68	IM1	CG18108	FBgn0034329	FBpp0085843	effector	2	2	2	0	2	1	1	1	2	1	1	0	par	par	IM2		
69	IM2	CG18106	FBgn0025583	FBpp0085845	effector	2	2	2	0	2	1	1	1	2	1	1	0	par	par	IM1	1.00	0.81
70	IM3	CG16844	FBgn0040736	FBpp0085846	effector	1	1	1	1	1	1	1	1	1	1	1	1	sco	sco		1.00	0.81
71	IM4	CG15231	FBgn0040653	FBpp0071487	effector	1	1	1	1	1	1	1	1	1	1	1	1	sco	sco		1.00	0.81
72	IM10	CG18279	FBgn0033835	FBpp0086780	effector	2	1	1	1	1	1	1	1	1	1	2	1	sco	par		1.00	0.81
73	IM23	CG15066	FBgn0034328	FBpp0085908	effector	1	0	1	1	1	1	1	1	1	1	1	1	sco	sco		0.33	0.62
74	Jon25Bii	CG8869	FBgn0031654	FBpp0078691	protease	1	1	0	1	1	1	1	1	1	0	0	0	lr			0.23	0.48
75	Jon65Aii	CG6580	FBgn0035666	FBpp0076750	protease	1	1	1	1	1	1	0	1	1	0	0	0	lr			0.78	0.81
76	Jon65Aiii	CG6483	FBgn0035665	FBpp0076749	protease	1	1	1	1	1	1	1	1	1	1	1	1	sco	sco		0.00	0.00
77	lectin-24A	CG3410	FBgn0040104	FBpp0077228	recognition	1	1	1	0	0	0	0	0	0	0	0	0	lr			0.04	0.14
78	Mtk	CG8175	FBgn0014865	FBpp0086518	effector	1	1	1	1	1	1	1	1	1	0	1	1	sco	sco		1.00	0.81
79	mthl2	CG17795	FBgn0035623	FBpp0076796	effector	4	4	7	5	6	1	1	1	4	1	1	2	par		Mthl1_3_4		
80	nec	CG1857	FBgn0002930	FBpp0088123	signalling	1	1	1	1	1	1	1	1	1	1	1	1	sco	sco		0.01	0.04
81	pll	CG5974	FBgn0010441	FBpp0084549	signalling	1	2	1	1	1	1	1	1	1	1	1	1	sco	sco		0.97	0.81
82	Pxn	CG12002	FBgn0011828	FBpp0072827	effector	1	1	2	1	1	1	1	1	1	1	2	1	sco			1.00	0.81
83	PGRP-LB	CG14704	FBgn0037906	FBpp0081872	recognition	1	1	1	1	1	1	1	1	1	1	1	1	sco	sco		1.00	0.81
84	PGRP-SA	CG11709	FBgn0030310	FBpp0073358	recognition	1	1	1	1	1	1	1	1	1	1	1	1	sco	sco		0.84	0.81
85	PGRP-SB1	CG9681	FBgn0043578	FBpp0075107	recognition	1	1	1	1	1	1	1	1	1	1	1	1	sco	sco		1.00	0.81
86	PGRP-SD	CG7496	FBgn0035806	FBpp0076519	recognition	1	1	2	1	1	1	1	1	1	1	1	1	sco	sco		1.00	0.81
87	Rel	CG11992	FBgn0014018	FBpp0088375	signalling	1	1	1	1	1	1	1	1	1	1	1	1	sco	sco		0.19	0.45
88	santa-maria	CG12789	FBgn0025697	FBpp0079088	recognition	1	1	1	1	1	1	1	1	1	1	1	1	sco	sco		1.00	0.81
89	Sp7	CG3066	FBgn0037515	FBpp0081237	signalling	1	1	2	1	1	1	1	1	1	1	1	1	sco	par		0.08	0.23
90	SPE	CG16705	FBgn0039102	FBpp0083832	signalling	1	2	2	2	2	1	1	1	1	1	1	1	par	sco			
91	spheroide	CG9675	FBgn0030774	FBpp0074137	signalling	1	1	1	1	1	1	1	1	1	1	1	1	sco	sco		0.15	0.37
92	spirit	CG2056	FBgn0030051	FBpp0071174	signalling	1	1	1	1	1	2	1	1	1	2	1	1	sco	par		1.00	0.81
93	Stam	CG6521	FBgn0027363	FBpp0079732	signalling	1	1	1	1	1	1	1	1	1	1	1	1	sco	sco		0.64	0.81
94	Stat92E	CG4257	FBgn0016917	FBpp0088487	signalling	1	1	1	1	1	1	1	1	1	1	1	1	sco	sco		1.00	0.81
95	Tep1	CG18096	FBgn0041183	FBpp0080369	recognition	1	1	1	1	1	1	0	0	0	0	0	0	rec_par	lr	TepII_III_IV_V	0.00	0.00
96	Tep2	CG7052	FBgn0041182	FBpp0079133	recognition	5	4	5	5	6	4	4	4	3	6	3	5	par	par	TepI_III_IV_V	0.00	0.00
97	Tep4	CG10363	FBgn0041180	FBpp0080795	recognition	5	4	5	5	6	4	4	4	3	6	3	5	par	par	TepI_II_III_V	0.00	0.00
98	Tequila	CG4821	FBgn0023479	FBpp0076252	protease	1	2	1	1	1	1	2	2	1	2	2	1	par			0.37	0.69
99	Tl	CG5490	FBgn0003717	FBpp0084431	signalling	1	1	1	1	1	1	1	1	1	1	1	1	sco	sco		1.00	0.81
100	TotA	CG31509	FBgn0028396	FBpp0083378	effector	1	2	2	1	2	0	0	0	0	0	0	0	lr	lr		0.96	0.81
101	TotB	CG5609	FBgn0038838	FBpp0083380	effector	1	1	1	2	0	0	0	0	0	0	0	0	lr	lr		0.07	0.21
102	TotC	CG31508	FBgn0044812	FBpp0083379	effector	1	1	1	0	0	0	0	0	0	0	0	0	lr	lr		1.00	0.81
103	epsilonTry	CG18681	FBgn0010425	FBpp0087256	protease	1	1	1	1	1	1	1	1	1	1	1	1	sco			0.22	0.47
104	lambdaTry	CG12350	FBgn0043470	FBpp0087222	protease	1	1	1	1	1	1	1	1	1	1	1	1	sco			1.00	0.81
105	yellow-c	CG4182	FBgn0041713	FBpp0080260	effector	1	1	1	1	1	1	1	1	1	1	1	1	sco			0.20	0.45
106	yellow-f	CG18550	FBgn0041710	FBpp0082191	effector	1	1	1	1	1	0	0	0	0	0	0	0	rec_par	par	Yellow-f2	0.00	0.00
107	yellow-f2	CG8063	FBgn0038105	FBpp0082190	effector	2	2	2	2	2	1	1	1	2	1	1	1	par	par	Yellow-f		
108	yellow-g	CG5717	FBgn0041709	FBpp0072780	effector	1	1	1	1	1	1	1	1	1	1	1	1	sco			1.00	0.81
109	alphaPS4	CG16827	FBgn0034005	FBpp0086503	recognition	1	1	1	1	1	0	0	0	0	0	0	0	rec_par		AlphaPS3_5	0.00	0.00
110	Adgf-A	CG5992	FBgn0036752	FBpp0089360	hemocyte	1	1	1	2	1	1	1	1	1	1	1	1	sco			0.44	0.79
111	dpp	CG9885	FBgn0000490	FBpp0077451	hemocyte	1	1	1	1	1	1	1	1	1	1	1	1	sco	sco		0.00	0.00
112	brm	CG5942	FBgn0000212	FBpp0075278	hemocyte	1	1	1	1	1	1	1	1	1	1	1	2	sco	sco			
113	phl	CG2845	FBgn0003079	FBpp0070385	hemocyte	1	1	1	1	1	1	1	1	1	1	1	1	sco	sco		0.32	0.62
114	srp	CG3992	FBgn0003507	FBpp0082669	hemocyte	1	1	1	1	1	1	1	1	1	1	1	1	sco			1.00	0.81
115	zfh1	CG1322	FBgn0004606	FBpp0085063	hemocyte	1	1	2	1	1	1	1	1	1	1	1	1	sco				
116	Su(H)	CG3497	FBgn0004837	FBpp0080261	hemocyte	1	1	1	1	1	1	1	2	1	1	1	1	sco	sco		0.07	0.21
117	Ser	CG6127	FBgn0004197	FBpp0084498	hemocyte	1	1	1	1	1	1	1	2	1	1	1	1	sco	sco		0.00	0.00
118	lwr	CG3018	FBgn0010602	FBpp0077741	hemocyte	1	1	1	1	1	1	1	1	1	1	1	1	sco	sco		1.00	0.81
119	gcm2	CG3858	FBgn0019809	FBpp0079435	hemocyte	1	1	1	1	1	1	1	1	1	1	1	1	sco	sco		1.00	0.81
120	wg	CG4889	FBgn0004009	FBpp0079060	hemocyte	1	0	1	1	1	1	1	1	1	1	1	1	sco			0.75	0.81
121	Nc	CG8091	FBgn0026404	FBpp0076076	hemocyte	1	1	1	1	1	1	1	1	1	1	1	1	sco			0.65	0.81
122	N	CG3936	FBgn0004647	FBpp0070483	hemocyte	1	1	1	1	1	1	1	1	1	1	1	1	sco	sco		0.65	0.81
123	gcm	CG12245	FBgn0014179	FBpp0079451	hemocyte	1	1	1	1	1	1	1	1	1	1	1	1	sco	sco		0.92	0.81
124	Pvf2	CG13780	FBgn0031888	FBpp0079051	hemocyte	1	1	1	1	1	1	1	1	1	1	1	1	sco	sco		0.12	0.31
125	Dsor1	CG15793	FBgn0010269	FBpp0071248	hemocyte	1	1	1	1	1	1	1	1	1	1	1	1	sco	sco		0.12	0.31
126	lz	CG1689	FBgn0002576	FBpp0071255	hemocyte	1	1	1	1	1	1	1	1	1	1	1	1	sco	sco		1.00	0.81
127	ytr	CG18426	FBgn0021895	FBpp0072143	hemocyte	1	1	1	1	1	1	1	1	1	1	1	1	sco	sco		1.00	0.81
128	Rac1	CG2248	FBgn0010333	FBpp0072614	hemocyte	1	1	1	1	1	1	1	1	1	1	1	1	sco	sco		1.00	0.81
129	l(3)mbn	CG12755	FBgn0002440	FBpp0076770	hemocyte	1	1	1	1	1	1	1	1	1	1	1	1	sco			0.15	0.37
130	mxc	CG12124	FBgn0260789	FBpp0071267	hemocyte	1	0	1	1	1	1	1	1	1	0	1	1	sco			0.32	0.62
131	Pvr	CG8222	FBgn0032006	FBpp0079244	hemocyte	1	1	1	1	1	1	1	1	1	1	1	1	sco			0.07	0.21

	Name	CG	Fbgn	Fbpp	Immune	mel	sim	sec	yak	ere	ana	per	pse	wil	moj	vir	gri	Homology	Sackton	paralog_to	M7vsM8	FDR
132	kn	CG10197	FBgn0001319	FBpp0086595	hemocyte_reg	1	1	1	1	1	1	1	1	1	1	1	1	sco	sco		0.60	0.81
133	Ras85D	CG9375	FBgn0003205	FBpp0081600	hemocyte_reg	1	1	1	1	1	1	1	1	1	1	1	1	sco	sco		0.05	0.17
134	Egfr	CG10079	FBgn0003731	FBpp0071570	hemocyte_reg	1	1	1	1	1	1	1	1	1	1	1	1	sco	sco		0.81	0.81
135	ush	CG2762	FBgn0003963	FBpp0077723	hemocyte_reg	1	1	1	1	1	1	1	1	1	1	1	1	sco	sco		0.00	0.00
136	RpS6	CG10944	FBgn0261592	FBpp0071087	hemocyte_reg	1	1	1	1	1	1	1	1	1	1	1	1	sco	sco		0.21	0.46
137	cher	CG3937	FBgn0014141	FBpp0088480	hemocyte_reg	1	1	1	1	1	1	1	1	1	2	2	1	sco	sco		0.00	0.00
138	dom	CG9696	FBgn0020306	FBpp0071528	hemocyte_reg	1	2	1	1	1	1	2	1	1	1	1	1	sco	sco			
139	edl	CG15085	FBgn0023214	FBpp0085834	hemocyte_reg	1	1	1	1	1	1	1	1	1	1	1	1	sco	sco		1.00	0.81
140	pnt	CG17077	FBgn0003118	FBpp0088656	hemocyte_reg	1	1	1	1	1	1	1	1	0	1	1	1	sco	sco		0.38	0.69
141	sgg	CG2621	FBgn0003371	FBpp0070449	hemocyte_reg	1	0	1	1	1	1	1	1	1	1	1	1	sco			0.00	0.00
142	Cyt-b5	CG2140	FBgn0264294	FBpp0087979	hemocyte_reg	1	1	1	1	1	1	1	1	1	1	1	1	sco			1.00	0.81
143	chn	CG11798	FBgn0015371	FBpp0110305	hemocyte_reg	1	1	1	1	1	1	1	1	1	1	1	1	sco			1.00	0.81
144	He	CG31770	FBgn0028430	FBpp0080189	hemocyte_reg	1	1	1	1	0	0	0	0	0	0	0	0	lr	lr		0.69	0.81

Each row contains the annotation of the gene (CG, FBpp and FBgn), the immune classification (Immune), the number of copies in each species (species abbreviation), the homology classification (Homology), the classification in [2], to which other gene is paralog (paralog to), the p-value of the likelihood test in PAML (M7vs M8) and the FDR.

Table S4. Lineage restricted genes

	Effector					Protease														Recognition			
	TotA	TotB	TotC	PPO3	yellow-f*	Spn88Eb *	CG11313 *	CG6639 *	CG30414 *	CG4259 *	CG12951 *	CG18477 *	Jon65Aii*	Jon25Bii	CG30090 *	CG18563	CG4793	CG3117	CG18478	lectin-24A	αPS4*	He	TepI *
mel	1	1	1	1	1	1	1	1	2	1	1	2	1	1	1	1	1	1	2	1	1	1	1
sim	2	1	1	1	1	1	1	1	1	1	1	1	1	1	1	1	1	1	0	1	1	1	1
sec	2	1	1	1	1	1	1	1	1	1	1	1	1	0	1	0	1	1	0	1	1	1	1
yak	1	2	0	1	1	1	1	1	1	1	1	1	1	1	1	1	1	1	1	0	1	1	1
ere	2	0	0	1	1	1	3	0	1	1	1	1	1	1	1	0	1	0	0	0	1	0	1
ana	0	0	0	0	0	0	0	0	0	1	1	2	1	1	1	0	0	0	0	0	0	0	1
per	0	0	0	0	0	0	0	1	0	0	0	0	0	1	0	0	1	0	0	0	0	0	0
pse	0	0	0	0	0	0	0	2	0	0	0	0	1	1	1	0	1	0	0	0	0	0	0
wil	0	0	0	0	0	0	0	0	0	0	0	0	1	1	1	0	0	0	0	0	0	0	0
moj	0	0	0	0	0	0	0	0	0	0	0	0	0	0	0	0	0	0	0	0	0	0	0
vir	0	0	0	0	0	0	0	0	0	0	0	0	0	0	0	0	0	0	0	0	0	0	0
Pathway	JakStat			PPO		non-identified																	JakStat

* Genes under positive selection

For all lineage restricted genes, the numbers of orthologs in each species is given according to the OrthoMCL algorithm [1]. From the 26 LR found, 23 are listed in this table and the remaining 3 are duplications inside *D. melanogaster* (table S3).

Table S5. List of primers for RT-qPCR

Amplicon name	Direction	Sequence 5 → 3′	Amplicon size (bp)
PPO3	F	AAGAGGATAAGGTTGACCAGAC	251
	R	GCTTCTCTTATTCAGGAGCGA	251
IM1	F	TCCACTGTCGCCCGATCC	92
	R	CTTGGGTTGAAACTTCCTACTTGC	92
FH68A	F	GAGCAGAAGAGCCCCTACCT	84
	R	AATGAAACCCTGACGTGGAC	84
ACT5C	F	CACACCAAATCTTACAAAATGTGT	83
	R	AATCCGGCCTTGCACATG	83
TEP1 *D. sechellia*	F	ACTAAGAAGTGGCTGTTATACC	190
	R	AGTTGGTGGTAATAAAGAACGG	190
TEP1 *D. simulans*	F	AAGATGAGTTAGCAAAGAACTCAG	282
	R	AAGTTGGTGGTAATAAAGAACGG	282
TEPI *D. melanogaster*	F	AGTCCCATAAAGGCCGACTGA	101
	R	CACCTGCATCAAAGCCATATTG	101

References

1. L. Li, C.J. Stoeckert, and D.S. Roos. Orthomcl: identification of ortholog groups for eukaryotic genomes. *Genome research*, 13(9):2178, 2003.

2. Timothy B Sackton, Brian Lazzaro, Jay D Evans, Hultmark Dan, and Andrew G Clark. Dynamic evolution of the innate immune system in *Drosophila. Nature genetics*, 39:1461–1468, 2007.

3. Todd A. Schlenke, Jorge Morales, Shubha Govind, and Andrew G. Clark. Constrasting infection strategies in generalist and specialist wasp parasitoids of *Drosophila melanogaster. Plos pathogens*, 3, 2007.

4. Bregje Wertheim, Alex R Kraaijeveld, Eugene Schuster, Eric Blanc, Meirion Hopkins, Scott D Pletcher, Michael R Strand, Linda Partridge, and H charles J Godfray. Genome-wide gene expression in response to parasitoid attack in *Drosophila. Genome Biology*, 6:1–20, 2005.

5. Z Yang and R. Nielsen. Estimating synonymous and nonsynonymous substitution rates under realistic evolutionary models. *Mol.Bio.Evol*, 17:32–43, 2000.
